# Novel computer aided diagnostic models on multimodality medical images to differentiate well differentiated liposarcomas from lipomas approached by deep learning methods

**DOI:** 10.1186/s13023-022-02304-x

**Published:** 2022-04-07

**Authors:** Yuhan Yang, Yin Zhou, Chen Zhou, Xuelei Ma

**Affiliations:** 1 Department of Pediatric Surgery, West China Hospital, Sichuan University, No. 37 Guoxue Alley, Chengdu, 610041 Sichuan China; 2 Department of Biotherapy and Cancer Center, State Key Laboratory of Biotherapy, West China Hospital, Sichuan University, No. 37 Guoxue Alley, Chengdu, 610041 China

**Keywords:** Lipoma, Well differentiated liposarcoma, Deep learning, Convolutional neural network, Magnetic resonance imaging, Computed tomography, Multimodality model

## Abstract

**Background:**

Deep learning methods have great potential to predict tumor characterization, such as histological diagnosis and genetic aberration. The objective of this study was to evaluate and validate the predictive performance of multimodality imaging-derived models using computer-aided diagnostic (CAD) methods for prediction of MDM2 gene amplification to identify well-differentiated liposarcoma (WDLPS) and lipoma.

**Materials and methods:**

All 127 patients from two institutions were included with 89 patients in one institution for model training and 38 patients in the other institution for external validation between January 2012 and December 2018. For each modality, handcrafted radiomics analysis with manual segmentation was applied to extract 851 features for each modality, and six pretrained convolutional neural networks (CNNs) extracted 512–2048 deep learning features automatically. Extracted imaging-based features were selected via univariate filter selection methods and the recursive feature elimination algorithm, which were then classified by support vector machine for model construction. Integrated with two significant clinical variables, age and LDH level, a clinical-radiological model was constructed for identification WDLPS and lipoma. All differentiation models were evaluated using the area under the receiver operating characteristics curve (AUC) and their 95% confidence interval (CI).

**Results:**

The multimodality model on deep learning features extracted from ResNet50 algorithm (RN-DL model) performed great differentiation performance with an AUC of 0.995 (95% CI 0.987–1.000) for the training cohort, and an AUC of 0.950 (95% CI 0.886–1.000), accuracy of 92.11%, sensitivity of 95.00% (95% CI 73.06–99.74%), specificity of 88.89% (95% CI 63.93–98.05%) in external validation. The integrated clinical-radiological model represented an AUC of 0.996 (95% CI 0.989–1.000) for the training cohort, and an AUC of 0.942 (95% CI 0.867–1.000), accuracy of 86.84%, sensitivity of 95.00% (95% CI 73.06–99.74%), and specificity of 77.78% (95% CI 51.92–92.63%) in external validation.

**Conclusions:**

Imaging-based multimodality models represent effective discrimination abilities between WDLPS and lipoma via CAD methods, and might be a practicable approach in assistance of treatment decision.

**Supplementary Information:**

The online version contains supplementary material available at 10.1186/s13023-022-02304-x.

## Introduction

Lipomatous tumors are one of the most common soft tissue tumors that lipoma contributes high incidence to the benign tumors and liposarcoma represents highly observed frequency in the malignant spectrum [1]. Well differentiated liposarcoma (WDLPS) takes the largest part of liposarcoma with the characteristics of local aggressiveness and amplification of the MDM2 gene [1]. WDLPS has potential to progress into dedifferentiated liposarcoma (DDLPS) with aggravated malignancy and poor prognosis [1]. The therapeutic strategies are different between lipoma and WDLPS that lipomas are not needed to be excised in most cases, whereas WDLPS demands surgical resection in case of further progression [2]. Nowadays, the standard way to differentiate WDLPS from lipoma applies fluorescence in situ hybridization (FISH) in biopsy samples that amplification of the MDM2 gene is absent in lipoma, but present in WDLPS [1, 3, 4]. The whole diagnostic procedure is complex that biopsy increases risks of tumor spreading and sampling errors, and FISH spends lots of time until the definite diagnosis. Therefore, a noninvasive and easy-to-reach tool is warranted to differentiate lipoma and WDLPS for clinical practice.

Lipoma and WDLPS share too many similarities of clinical variables to distinguish each other, and there are limited imaging-associated differences in terms of size, location, tumor depth, and intra-tumor heterogeneity [5–9]. These indicators provide insufficient differentiation abilities to identify lipoma and WDLPS, but imaging materials have enormous information ready for exploration in association with tumor characterization. Advanced methods, innovative computer-aided diagnosis (CAD) techniques have potential for imaging-based classification, including feature-based and feature learning-based strategies [10]. The handcrafted radiomics, as the feature-based strategy, can explore underlying biological information from imaging materials by extracting high-throughput quantitative features on manual segmentation and interpreting each feature with corresponding tumor characteristics [11]. Deep learning methods, as an emerging field of feature learning-based strategy, represent improvement in recognizing images and consistency in imaging feature interpretation [12–14]. Multilayered convolutional neural networks (CNNs) on own database, as the conventional work-up in deep learning analysis, exist limitation in solving one specific clinical issue due to insufficient sample size for model training. Transfer learning methods have been applied to improve diagnostic accuracy on small-sample medical imaging by shifting pretrained CNN architecture to a new database [15, 16]. An alternative for transfer learning is to extract deep learning-derived features on pre-trained CNNs and classify these features with machine learning methods, which can achieved satisfactory performance in predictive accuracy and computational costs simultaneously for clinical tasks [17]. Therefore, an approach on imaging using CAD techniques might improve differentiation capacities between WDLPS and lipoma.

In this study, we hypothesized that multimodality (CT and MRI) deep learning methods could be implemented to evaluate amplification of MDM2 gene in order to classify WDLPS and lipoma. The objectives of our study were to apply advanced and noninvasive CAD tools on multimodality images to classify WDLPS and lipoma, validate their predictive abilities externally, and evaluate their efficiency comprehensively.

## Methods

### Patients

This retrospective study was approved by the Ethics Committee of the Institutional Review Board of two institutions, and patients’ consent was waived because of the retrospective design. All eligible patients underwent surgical resection of the whole tumor between January 2012 and December 2018, and received pre-operative multimodality imaging examinations with available contrast enhanced CT images and axial T1-weighted imaging (T1WI) and fat-suppressed T2-weighted imaging (T2FS) within one month before operation. All patients were diagnosed as lipoma or WDLPS with definite MDM2 amplification status via FISH test proven by histological evidence which were obtained from postoperative resected samples. The including criteria were listed as follow: (a) histologically confirmed as lipoma or WDLPS; (b) received surgical resection of primary lesions; (c) received pre-operative CT and MRI scanning. Patients were excluded if (a) having incomplete medical records; (b) having a history of other malignancies; (c) receiving anticancer treatments before the baseline CT or MRI scanning. Patients included from one institution were assigned to the training cohort for model training and cross validation, and patients from the other institution were assigned to the validation cohort for external validation. The detailed procedure of CT and MRI imaging acquisition was shown in Additional file 1: Supplementary Methods.

### Tumor segmentation

Two experienced radiologists (Z.H and D.W) delineated ROIs of the whole tumor on CT and MRI images manually using ITK-SNAP software [18]. The manual contoured ROIs of regions of interest (ROIs) was prepared for handcrafted radiomics features extraction. There were 20% patients from the training cohort selected randomly and blindly for repetitive segmentation of ROIs by two radiologists a week after the first segmentation. The intra-class correlation coefficients (ICC) were evaluated between first and second segmentation that only the radiomics features with ICC > 0.85 were considered for feature selection and model construction. The ROIs for deep learning analysis was completed by resizing the bounding box covering the radiologist-delineated ROIs into 224 mm * 224 mm and adjusting segmentation for tumor regions automatically, which were ready for input of pretrained CNNs for feature extraction.

### Deep learning features

Six pretrained deep learning CNN architectures were applied on CT, T1WI and T2FS images for extraction of representative deep learning features, including Xception [19], VGG16 [20], VGG19 [20], ResNet50 [21], InceptionV3 [22], and InceptionResNetV2 [23]. These pretrained CNNs were commonly used in the large-scale and well-annotated ImageNet database [24]. We retained the convolutional base of pretrained CNNs, and extracted representative numeric values as representativeness of deep learning features from these CNNs regarding numbers of feature maps (2048 for ResNet50, InceptionV3 and Xception, 512 for VGG16 and VGG19, and 1536 for InceptionResNetV2). Detailed description about how deep learning features were acquired from pretrained CNNs was shown in Additional file 1: Methods. These extracted deep learning features from different pre-trained CNNs would be ready for features selection and model construction via the machine learning approaches at the next step. Regarding to the unclear mechanism of deep learning features for classification of clinical outcomes, Gradient-weighted Class Activation Mapping (Guided Grad-CAM) was applied to highlight specific subregions identified by CNNs for generation of deep learning features by visualizing the output of CNNs at the last convolutional layer [25].

### Handcrafted radiomics features

Using an automated mode of PyRadiomics package (version 2.1.2), handcrafted radiomics features with or without wavelet were extracted on the radiologist-drawn ROIs of CT and MRI images, respectively [26]. The study design complied with the image biomarker standardization initiative (IBSI) reporting guidelines. Radiological associated biomarker consisted of three categories: (a) first-order statistics; (b) shape features; (c) second-order statistics: gray level co-occurrence matrix (GLCM), gray level run length matrix (GLRLM), gray level size zone matrix (GLSZM), gray level dependence matrix (GLDM), and neighborhood gray tone difference matrix (NGTDM). Most of radiomics features mentioned above showed consistency with feature definitions in accordance with the IBSI guidelines [27–29] with additional explanations in Additional file 1: Supplementary Methods.

### Feature selection and differentiation model construction

Both handcrafted radiomics and deep learning features were used to construct imaging-associated models in differentiation of lipoma and WDLPS by machine learning approaches. A previous study has evaluated various machine learning combination of feature selection and model construction with the area under the receiver operating characteristic curve (AUC) [30]. We adopted the optimal combination with the largest AUC among all combinations as the machine learning approach in this study. The sklearn.feature_selection module of Scikit-Learn package was implemented for feature selection, including univariate filter selection methods and the recursive feature elimination algorithm. The top 20% best features calculated for prediction of MDM2 amplification were selected based on F-test estimate the degree of linear dependency between two random variables in the univariate filter selection. Next, a wrapper feature selection method called the recursive feature elimination algorithm was used to select the predictive features by recursively considering smaller and smaller sets of features. First, the estimator is trained on the initial set of features and the importance of each feature is obtained either through any specific attribute or callable. Then, the least important features are pruned from current set of features. That procedure is recursively repeated on the pruned set until the desired number of features to select is eventually reached. We used the support vector machine (SVM) with a radial basis kernel function as a classifier for model construction [31]. For differentiation model construction and unbiased performance evaluation, optimal parameters for image pre-processing and training were identified using 10 iterations of five-fold nested cross-validation. The details of differentiation model construction were shown in Additional file 1: Supplementary Methods.

The imaging-associated model with the highest AUC value during cross validation was chosen to generate a radiological signature. All clinical variables were evaluated by logistic regression analysis that the univariate analysis selected the variables with p value less than 0.10 to enter the multivariate regression model. Only the clinical variables with p value less than 0.05 in the multivariate analysis were considered as significant clinical variables. A clinical-radiological nomogram model was developed integrating the radiological signature and significant clinical variables. For comparison, a clinical model was constructed based on significant clinical variables only via logistic regression method.

### Statistical analysis

The distribution differences of clinical variables between the training and validation cohorts were evaluated by Fisher’s exact tests or Chi-square tests for categorical data and non-parametric Mann–Whitney test for continuous data. To evaluate predictive performance of differential models, we applied the receiver operating characteristic (ROC) curves and their AUC value, and precision-recall plots and their average precision (AP) value, respectively. Accuracy, sensitivity, specificity, positive predictive value (PPV), and negative predictive value (NPV) and their 95% confidence intervals (CI) were calculated from confusion matrix as measurement indicators to assess predictive models quantitatively. The calibration performance was measured by the Hosmer–Lemeshow test and visualized by calibration plots [32]. Discrimination ability was quantified by Harrell's concordance indices (C-index) [33]. The clinical usefulness of the integrated differential model was evaluated by the net benefit of decision curve analysis [34].

A two-tailed P value less than 0.05 was considered as statistical significance. The whole statistical analysis and graphic production were completed by Python (version 3.8) and R (version 3.6.1). All involved packages in this study are listed in Additional file 1: Supplementary Methods.

## Results

### Patients’ characteristics

**Fig. 1:**
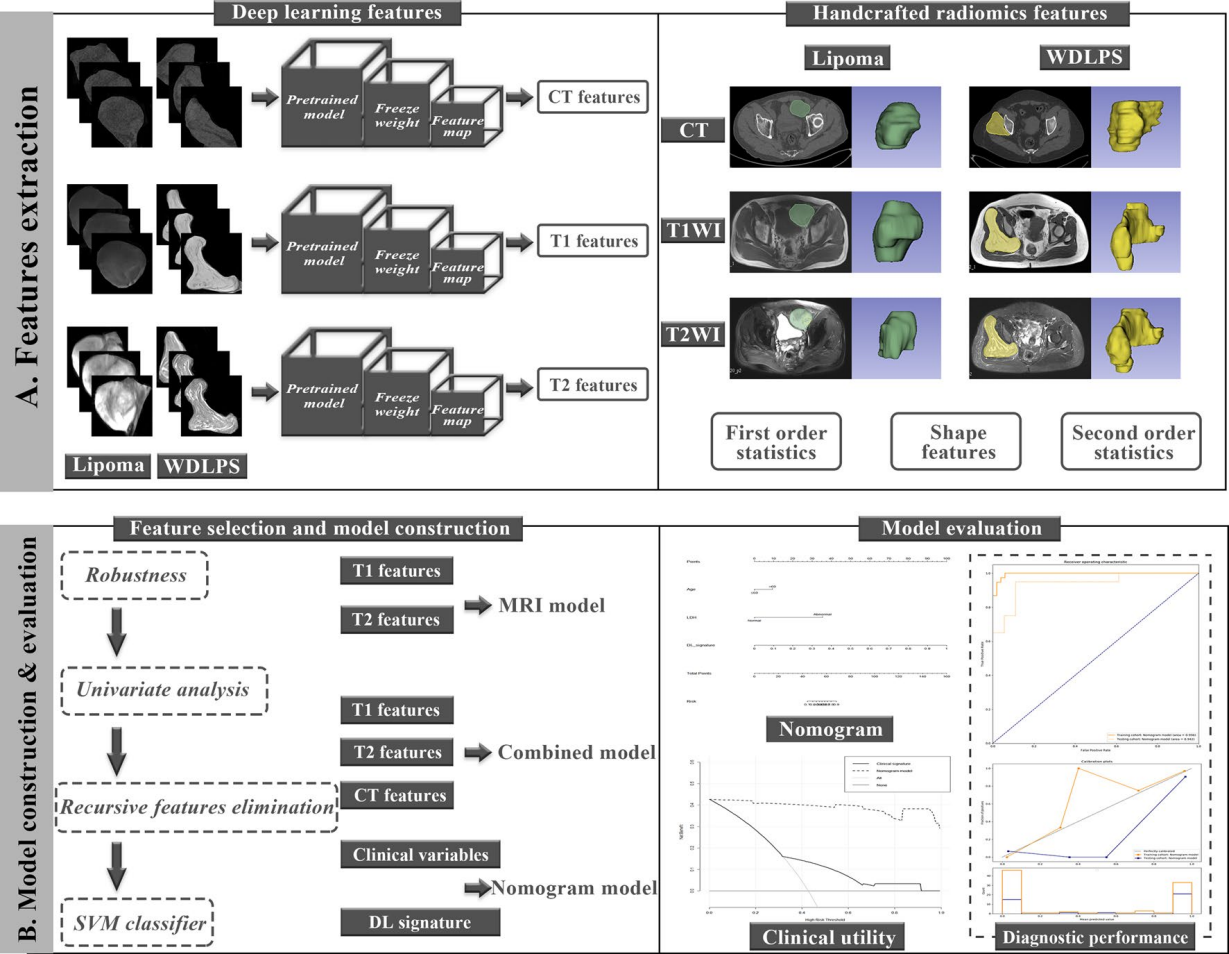
A general flowchart of data analysis. **A** Imaging-derived features were extracted by the deep learning analysis and handcrafted radiomics analysis on multimodality medical images, including CT and MRI, respectively. **B** Predictive models on both deep learning and handcrafted radiomics features for classification of lipoma and WDLPS were approached by machine learning methods including features selection and model construction. The deep learning-based model with the optimal performance was chosen to generate a deep learning signature. An integrated differentiation model was constructed by the deep learning signature and independent clinical predictors. All differentiation models were evaluated by ROC curves, precision-recall plots, and calibration plots in both training and validation cohorts. CT, Computed tomography; MRI, Magnetic resonance imaging; WDLPS, Well-differentiated liposarcoma; ROC, Receiver operating characteristic; SVM, Support vector machine

The whole scheme was depicted including the radiological feature extraction, and diagnostic model construction and evaluation (Fig. 1). A total of 127 patients were included from two institutions in this study that 89 patients from one institution were assigned to the training cohort, and 38 patients from the other institution were assigned to the external validation cohort. Detailed demographic and laboratory characteristics were shown in Table 1. There were no significant differences found between clinical variables in the training and testing cohorts that the baseline characteristics of two institutions represented similar distribution without statistical significance (P > 0.05). No significant difference in the distribution of lipoma or WDLPS was identified between the training and external validation cohorts (P = 0.303). Age at diagnosis (p = 0.009) and lactate dehydrogenase (LDH) level (p = 0.050) represented their predictive abilities for differentiation of lipoma and WDLPS significantly in the training cohort (Additional file 1: Table S1). This clinical differentiation model yielded an AUC of 0.652 (95% CI 0.534–0.770 versus 0.504 (95% CI 0.318–0.690), and an accuracy of 65.17% versus 50.00% for the training and validation datasets, respectively (Table 2 and Additional file 1: Fig. S1).

**Table 1:** Demographic characteristics of patients in the training and testing cohorts

Characteristic	All subjects N = 127)	Training cohort (N = 89)	Validation cohort (N = 38)	*P* value
**Diagnosis, no. (%)**				0.303
Lipoma	69 (54.3)	51 (57.3)	18 (47.4)	
WDLPS	58 (45.7)	38 (42.7)	20 (52.6)	
**Gender, no. (%)**				0.116
Female	60 (47.2)	38 (42.7)	22 (57.9)	
Male	62 (52.8)	51 (57.3)	16 (42.1)	
**Age, median (IQR), years**	48 (23)	48 (23)	49 (19)	0.904
≤ 60, no. (%)	98 (77.2)	69 (77.5)	29 (76.3)	0.882
> 60, no. (%)	29 (22.8)	20 (22.5)	9 (23.7)	
**Tumor size, median (IQR), cm**	7.0 (6.4)	7.0 (6.6)	7.3 (7.9)	0.602
≤ 10, no. (%)	85 (66.9)	59 (66.3)	26 (68.4)	0.815
> 10, no. (%)	42 (33.1)	30 (33.7)	12 (31.6)	
**Tumor location, no. (%)**				0.140
Extremity	61 (48.0)	43 (48.3)	18 (47.4)	
Trunk	41 (32.3)	25 (28.1)	16 (42.1)	
Abdomen/retroperitoneal	25 (19.7)	21 (23.6)	4 (10.5)	
**Tumor depth, no. (%)**				0.228
Superficial	15 (11.8)	13 (14.6)	2 (5.3)	
Deep	112 (88.2)	76 (85.4)	36 (94.7)	
**HGB**^**a**^**, median (IQR), g/L**	136 (22)	138 (27)	134 (17)	0.383
Normal, no. (%)	104 (81.9)	71 (79.8)	5 (13.2)	0.344
Abnormal, no. (%)	23 (18.1)	18 (20.2)	33 (86.8)	
**Platelet, median (IQR), 10**^**9**^**/L**	188 (84)	196 (79)	185 (100)	0.992
Normal (≤ 300), no. (%)	111 (87.4)	79 (88.8)	32 (84.2)	0.561
Abnormal (> 300), no. (%)	16 (12.6)	10 (11.2)	6 (15.8)	
**WBC, median (IQR), 10**^**9**^**/L**	6.26 (2.53)	6.05 (2.42)	6.52 (2.26)	0.218
Normal (> 4), no. (%)	108 (85.0)	79 (88.8)	29 (76.3)	0.101
Abnormal (≤ 4), no. (%)	19 (15.0)	10 (11.2)	9 (23.7)	
**ALB, median (IQR), g/L**	42.7 (4.8)	42.5 (4.6)	43.7 (5.5)	0.183
Normal (> 40), no. (%)	98 (77.2)	68 (76.4)	30 (78.9)	0.755
Abnormal (≤ 40), no. (%)	29 (22.8)	21 (23.6)	8 (21.1)	
**ALP, median (IQR), U/L**	76 (42)	73 (42)	80 (48)	0.517
Normal (≤ 140), no. (%)	116 (91.3)	82 (92.1)	34 (89.5)	0.732
Abnormal (> 140), no. (%)	11 (8.7)	7 (7.9)	4 (10.5)	
**LDH, median (IQR), U/L**	160 (46)	155 (38)	167 (57)	0.052
Normal (≤ 220), no. (%)	110 (86.6)	80 (89.9)	30 (78.9)	0.152
Abnormal (> 220), no. (%)	17 (13.4)	9 (10.1)	8 (21.1)	
**Duration of hospitalization, median (IQR), day**	10 (10)	10 (11)	10 (9)	0.960
≤ 14, no. (%)	87 (68.5)	59 (66.3)	28 (73.7)	0.412
> 14, no. (%)	40 (31.5)	30 (33.7)	10 (26.3)	

WDLPS, Well-differentiated liposarcoma; IQR, Interquartile range; HGB, Hemoglobin; WBC, White blood cell; ALB, Serum albumin; ALP, Alkaline phosphatase; LDH, Lactate dehydrogenase

^a^For male, HGB < 130 is defined abnormal; for female, HGB < 115 is defined abnormal

**Table 2:** Predictive performance of significant clinical variables, the deep learning signature, and clinical and clinical-deep learning models in classification of WDLPS and lipoma on patients in the training and validation cohorts

Parameters	Training cohort						Validation cohort					
	AUC	Accuracy	Sensitivity	Specificity	PPV	NPV	AUC	Accuracy	Sensitivity	Specificity	PPV	NPV
*Variables*												
Age	0.625 (0.505–0.746)	66.29 (59/89)	36.84 (22.29–54.00)	88.24 (75.44–95.13)	70.00 (45.67–87.16)	65.22 (52.71–76.02)	0.514 (0.328–0.700)	50.00 (19/38)	25.00 (9.59–49.41)	77.78 (51.92–92.63)	55.56 (22.65–84.66)	48.28 (29.89–67.10)
LDH	0.572 (0.450–0.695)	62.92 (56/89)	18.42 (8.32–34.89)	44.09 (85.41–99.32)	77.78 (40.19–96.05)	61.25 (49.67–71.74)	0.436 (0.251–0.622)	42.11 (16/38)	15.00 (3.96–38.86)	72.22 (46.41–89.29)	37.50 (10.24–74.11)	43.33 (25.98–62.34)
DL signature^a^	0.995 (0.987–1.000)	95.51 (85/89)	92.11 (77.52–97.94)	98.04 (88.21–99.90)	97.22 (83.80–99.85)	94.34 (83.37–98.53)	0.950 (0.886–1.000)	92.11 (35/38)	95.00 (73.06–99.74)	88.89 (63.93–98.05)	90.48 (68.17–98.33)	94.12 (69.24–99.69)
*Models*												
Clinical model^b^	0.652 (0.534–0.770)	65.17 (58/89)	39.47 (24.49–56.55)	84.31 (70.86–92.52)	65.22 (42.82–82.81)	65.15 (52.34–76.19)	0.504 (0.318–0.690)	50.00 (19/38)	40.00 (19.98–63.59)	61.11 (36.14–81.74)	53.33 (27.42–77.72)	47.83 (27.42–68.92)
Clinical-DL model^c^	0.996 (0.989–1.000)	95.51 (85/89)	97.37 (84.57–99.86)	94.12 (82.77–98.47)	92.50 (78.52–98.04)	97.96 (87.76–99.89)	0.942 (0.867–1.000)	86.84 (33/38)	95.00 (73.06–99.74)	77.78 (51.92–92.63)	82.61 (60.45–94.28)	93.33 (66.03–99.65)

WDLPS, Well-differentiated liposarcoma; LDH, Lactate dehydrogenase; DL, Deep learning; AUC, Area under the receiver operating characteristic curve; PPV, Positive predictive value; NPV, Negative predictive value

^a^The deep learning signature was generated from the best deep learning model considering AUC of the validation cohort on different imaging examinations

^b^The clinical model was constructed by significant clinical variable, age at diagnosis and LDH selected by multivariate analysis with p value less than 0.05 in the training cohort

^c^The clinical-deep learning model was constructed by significant clinical variables, age at diagnosis and LDH, and the deep learning signature

### Differentiation model on deep learning analysis

**Fig. 2:**
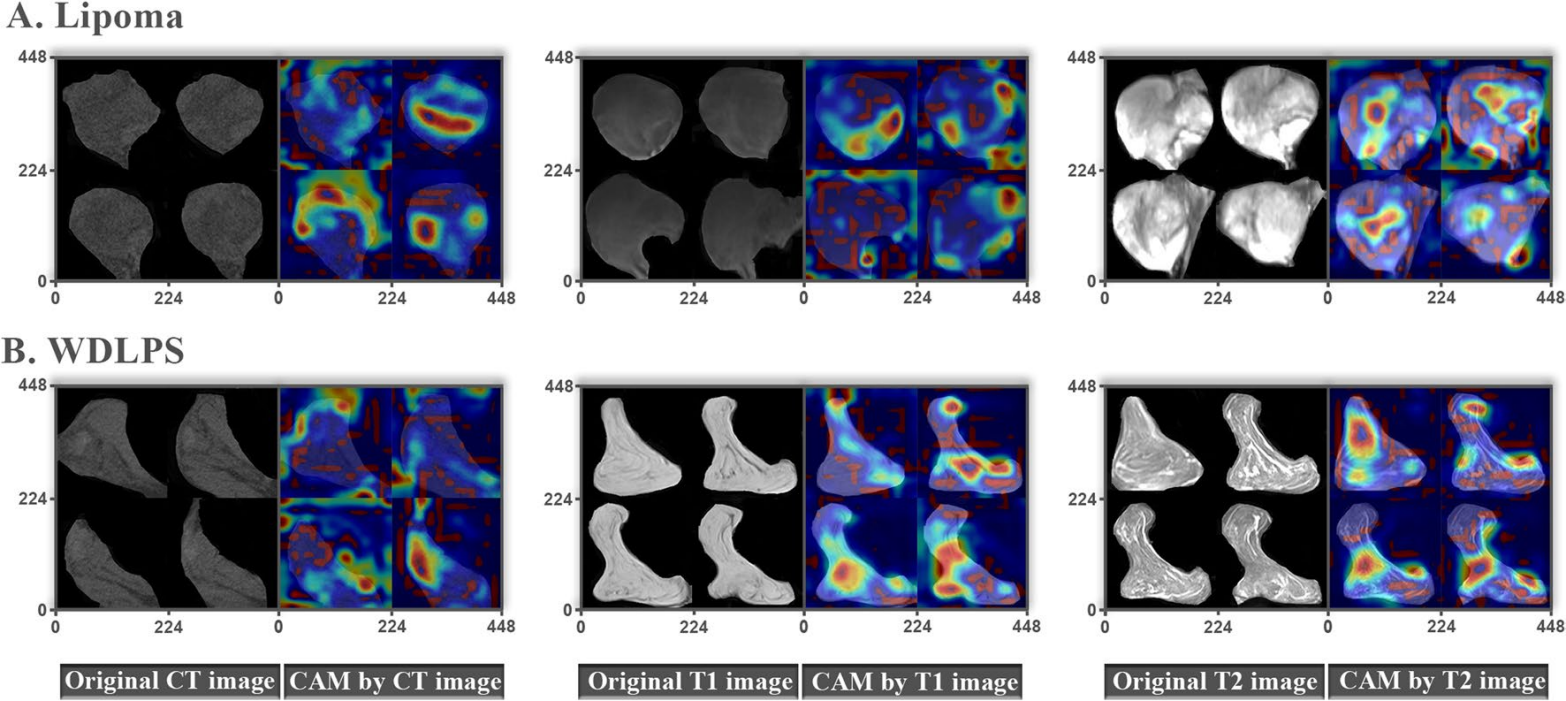
Feature heatmaps of representative patients on the deep learning ResNet50 algorithm via the Guided Grad-CAM. The original CT and MRI images and their corresponding feature heatmaps were shown from left to right. The red color highlighted the region of interest to classify lipoma and WDLPS. The red color focused on different area for lipomas (**A**) and WDLPS (**B**) on CT (Left), T1WI (Middle) and T2FS (Right) MRI images, respectively. CAM, Class activation mapping; CT, Computed tomography; MRI, Magnetic resonance imaging; WDLPS, Well differentiated liposarcoma; T1WI, T1-weighted MRI sequence; T2FS, Fat-saturated T2-weighted MRI sequence

For the differentiation models on deep learning features, thirty deep learning models, models were constructed, which were evaluated by measurement indicators and visualized by ROC curves and calibration plots for cross validation in the training cohort and for external validation in the validation cohort (Additional file 1: Tables S2, S3, and S4, Additional file 1: Figs. S2, S3, and S4). In all multimodality integrated models, the AUC ranged from 0.921 to 0.995 for the training group, and from 0.703 to 0.950 for the validation group (Additional file 1: Table S2). The multimodality model adopting ResNet50 architecture (RN-DL model) consisted of three MRI-derived and two CT-derived features, and achieved the best differentiation performance considering the AUC value in external validation among all radiological models with an AUC of 0.995 (95% CI 0.987–1.000), the C-index of 0.995, accuracy of 95.51%, sensitivity of 92.11% (95% CI 77.52–97.94%), specificity of 98.04% (95% CI 88.21–99.90%), PPV of 97.22% (95% CI 83.80–99.85%), NPV of 94.34% (95% CI 83.37–98.53%) in the training cohort, and an AUC of 0.950 (95% CI 0.886–1.000), the C-index of 0.950, accuracy of 92.11%, sensitivity of 95.00% (95% CI 73.06–99.74%), specificity of 88.89% (95% CI 63.93–98.05%), PPV of 90.48% (95% CI 68.17–98.33%), and NPV of 94.12% (95% CI 69.24–99.69%) in the validation cohort (Additional file 1: Table S2). The number of deep learning features selected for deep learning-based models was summarized in Additional file 1: Table S5. For ResNet50 CNN model, we evaluated the performance of differentiation models on deep learning features extracted from earlier layers of ResNet50 architecture. The results showed the greater performance of deep learning models on features from the last layer (Res5c) before the fully connected layer compared with over those on features from the earlier layers (Additional file 1: Table S6). The results verified the optimality of current feature extraction strategy on ResNet50 algorithm. Otherwise, as shown in feature maps, the valuable tumoral and peri-tumoral areas were highlighted by ResNet50 CNN interpretating important subregions for generation of the output features in further clinical view (Fig. 2).

### Differentiation model on handcrafted radiomics analysis

There were 851 features extracted from each modality (851 T1WI features and 851 T2FS features), including 107 features from original images, and 744 features from wavelet filtered images. Three T1WI-derived feature and four T2FS-derived features from all MRI radiomic features were selected to construct the MRI radiomics model, and these seven features integrated with two CT-derived features were used to generate a multimodality radiomics model (Additional file 1: Table S7). The multimodality radiomics model showed an AUC of 0.594 (95% CI 0.401–0.785), the C-index of 0.594, accuracy of 57.89%, sensitivity of 50.00% (95% CI 27.85–72.15%), specificity of 66.67% (95% CI 41.15–85.64%), PPV of 62.50% (95% CI 35.87–83.72%), and NPV of 54.55% (95% CI 32.67–74.93%) in external validation (Additional file 1: Table S2). The ROC curves, precision-recall plots, and calibration plots of the handcrafted radiomics models were shown in Additional file 1: Fig. S5.

### Clinical-radiological differentiation nomogram construction

**Fig. 3:**
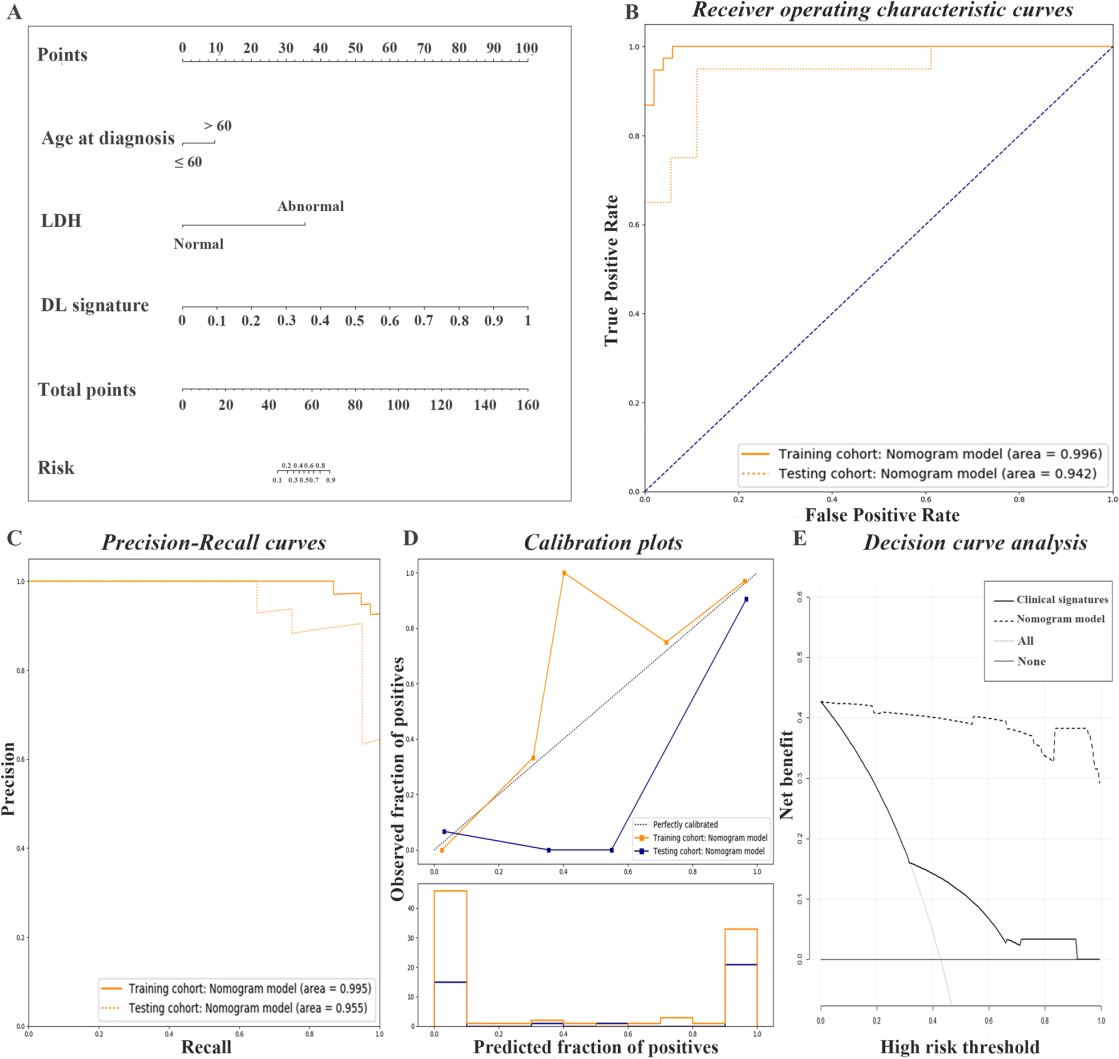
Evaluation of predictive performances for the integrated clinical-deep learning nomogram in classification of lipoma and WDLPS. **A** Nomogram model combining significant clinical variables, age at diagnosis and serum LDH level, and the deep learning signature. The deep learning signature was generated from the multimodality deep learning-based ResNet50 model with the largest AUC value among all models during external validation. **B** ROC curves for the predictive performance of the integrated clinical-deep learning nomogram in the training and validation cohorts, respectively. **C** Precision-recall plots for the predictive performance of the integrated clinical-deep learning nomogram in the training and validation cohorts, respectively. **D** Curves of the calibration analysis for the integrated clinical-deep learning nomogram in the training and validation cohorts, respectively. **E** The decision curve analysis for the integrated clinical-deep learning nomogram. WDLPS, Well-differentiated liposarcoma; AUC, Area under the receiver operating characteristic curve; ROC, Receiver operating characteristic

The deep learning signature was generated based on the multimodality RN-DL model considering AUC value in external validation. An integrated nomogram model was constructed in corporation with significant clinical variables, age at diagnosis and LDH level, and the deep learning signature (Fig. 3A). This nomogram model showed an AUC of 0.996 (95% CI 0.989–1.000), the C-index of 0.996, accuracy of 95.51%, sensitivity of 97.37% (95% CI 84.57–99.86%), specificity of 94.12% (95% CI 82.77–98.47%), PPV of 92.50% (95% CI 78.52–98.04%), and NPV of 97.96% (95% CI 87.76–99.89%) in the training cohort, and an AUC of 0.942 (95% CI 0.867–1.000), the C-index of 0.942, accuracy of 86.84%, sensitivity of 95.00% (95% CI 73.06–99.74%), specificity of 77.78% (95% CI 51.92–92.63%), PPV of 82.61% (95% CI 60.45–94.28%), and NPV of 93.33% (95% CI 66.03–99.65%) in the validation cohort (Table 2). The integrated model represented good differentiation performance in terms of ROC curves and precision-recall plots (Fig. 3B, C), and showed consistent calibration capacities (Fig. 3D) and satisfactory clinical benefit (Fig. 3E). There was a slight reduction of differentiation performance compared with the deep learning signature only considering the AUC value in external validation.

## Discussion

The present study developed diagnostic models for differentiation of WDLPS and lipoma receiving surgical resection based on the multimodality imaging-associated features by deep learning or handcrafted radiomics methods, and validated their predictive models in an independent cohort to assure their discriminative capacities. This study applied transfer learning technique for extraction of representative deep learning features from pre-trained CNNs. Both the multimodality deep learning-based model on ResNet50 algorithm (RN-DL model) and the integrated clinical-deep learning model achieved satisfactory discriminative abilities in classification of WDLPS and lipoma.

As our results shown, the present models trained on the imaging-based features represented greater prediction value than the model trained on the significant clinical variables, age at diagnosis and serum LDH level after external validation. The closer and clearer connection of imaging-based features connected with biological features was detected compared with the clinical variables. The multimodality-based models performed better generally than the unimodality-based models. The combination of different imaging modalities indicated improvement of prediction capacities for MDM2 amplification. In present study, integration of clinical variables and the imaging-based signature were tried to improve prediction abilities based on imaging-based signature only, but the results were unexpected that the optimal imaging-based model, RN-DL model (AUC: 0.950, 95% CI 0.886–1.000) performed better than the integrated model (AUC: 0.942, 95% CI 0.867–1.000) trained on the significant clinical variables and the deep learning signature in external validation. The integrated model showed slight differentiation improvement compared with the RN-DL multimodality model in cross validation process on the training cohort, but did not outperform the RN-DL multimodality model in external validation that the combination of clinical variables seemed not to improve the discriminative ability with little reduction for identification of WDLPS and lipoma. Considering the small-sample validation cohort, the real differentiation efficiency of clinical variables would be easily interfered with inevitable inter-cohort bias causing the unpredicted reduction of diagnostic efficiency. Otherwise, the present study based on one-institution external validation performed little compatibility considering the distribution of clinical variables with insufficient representativeness in the real-world clinical practice. Further validation was warranted for evaluating the efficiency and generality of the integrated clinical-radiological model with multi-institutional large-sample cohort. The imaging-only and integrated models had potential to become novel and convenient tools for clinicians and radiologists in classification of WDLPS and lipoma.

Handcrafted radiomics, as the feature-based strategy, is one of important aspects in CAD, which mainly focuses on extracting a large number of quantitative features from original images and constructing models to solve various clinical problems, for example classifying histological or molecular subtypes. In this study, the results were consistent with those from previous studies [35, 36] using quantitative radiomics analysis to differentiate WDLPS from lipoma. The present study showed highlights in aspects of the larger sample size and strict including criteria. The diagnostic models by Thornhill et al. included extensive range of liposarcoma subtypes such as DDLPS and myxoid liposarcoma to enlarge their sample size which might result in unpredictable errors that other liposarcoma subtypes showed distinct imaging-derived features easily identified by experienced radiologists [8, 37]. The differentiation of WDLPS and lipoma is more relevant to clinical practice and more urgent to be solved. The status of MDM2 amplification in present study was completed by FISH tests on resected sample, which assured the reliability of eligible subjects. The present imaging-based models took full advantage of routine imaging materials from the initial diagnosis, which performed favorable generalizability and feasibility than the standard FISH manner [1, 3, 4] in daily application.

The present study applied another CAD strategy, deep learning analysis to enhance the differentiation efficiency of constructed models and generalizability of utility of medical images. The pre-trained CNNs as feature extractors integrated machine learning approaches for model construction have provided feasible solutions for the weakness of conventional deep learning CNNs constructed and classified on our own datasets after translation of well-trained model parameters and promotion of the generalizability and plausibility in replication and validation [38–40]. Our results provided supporting evidence about the efficacy of transfer learning with the satisfactory results on multimodality medical images in prediction of MDM2 amplification. The operating procedure of these pre-trained CNNs was hard to interpret for understanding directly, so we used the visualized feature maps to translate the important subregions for feature generation into the highlighted area. These highlighted subregions might assist clinicians identifying image patterns. As shown in the feature heatmaps, the red color highlighted the region of interest to classify lipoma and WDLPS. The red color focused on different area of feature maps for lipoma and WDLPS that the marginal area was comparatively important for lipomas and the central area might contribute more in generation of deep learning features for WDLPS. The valuable tumoral and peri-tumoral areas peritumoral microenvironment might be useful for detection of spatial heterogeneity of tumoral and peritumoral microenvironment, which would be interpreted for further clinical view.

In this study, we constructed novel CAD models to optimize the differentiation performance in classification of lipoma and WDLPS. The CAD models could act as a reliable and cost-effective complementary tool based on conventional biopsy that the CAD classifier could be used to classify lipoma and WDLPS as a reference of therapeutic decisions in cases without biopsy specimens due to hard-to-reach anatomic location or contraindications or without the clear results of pathological and immunohistochemistry work-up in short time. For cases exhibiting inhomogeneous intra-tumoral subregions that are difficult to access targeted area, further development of CAD-based tools could assist identifying MDM2 amplification with assessment of the intact lesions to increase certainty in one-time pathological diagnosis.

There were some limitations existing in this study. First, there existed the heterogeneity between the source of pre-trained CNN bases and target databases, even though we adopted the transfer learning to mitigate it. The ideal solution of this problem is to develop large databases on specific clinical tasks with sufficient amounts of annotated medical images for training our own CNN model from scratch, which will represent excellent generalizability and clinical practicability. Second, the methods we applied to assure the feature robustness had some shortages that the ROI contour-based method might be slightly inferior to the test–retest imaging method for the feature repeatability [28, 41]. The test–retest method will be applied in a new prospective study our research team is performing to assure feature robustness. Last, the small-sample retrospective external validation resulted in inevitable bias and unexpected prediction performance. Our research group is performing a new study using our novel CAD models with a prospective large-sample cohort in multiple institutions.

In conclusion, this study constructed and validated novel CAD models via transfer learning technique on imaging materials in differentiation of WDLPS and lipoma receiving surgical excision. The multimodality deep learning-based models achieved the satisfactory differentiation performance, and represented a novel manner with high efficiency and without manual tumor delineation. Our CAD models could act as a reliable and cost-effective complementary tool based on pathological biopsy for reference of therapeutic decisions.

## Supplementary Information

**Additional file 1.** Supplementary Methods, Supplementary Tables, and Supplementary Figures.

## Data Availability

The datasets used and/or analyzed during the current study are available from the corresponding author, Xuelei Ma on reasonable request.

## Abbreviations

WDLPS: Well differentiated liposarcoma
CNN: Convolutional neural networks
DL-CNN: Deep learning-convolutional neural networks
CT: Computed tomography
MRI: Magnetic resonance imaging
T1WI: T1-weighted imaging
T2FS: Fat-suppressed T2-weighted imaging
AJCC: American Joint Committee on Cancer
TR: Repetition time
TE: Echo time
CAM: Class activation mapping
ROI: Region of interest
IQR: Interquartile range
AUC: Area under the receiver operating characteristic curve
PPV: Positive predictive value
NPV: Negative predictive value
ROC: Receiver operating characteristic
CAD: Computer-aided diagnosis
