# Supplementary material for: Novel computer aided diagnostic models on multimodality medical images to differentiate well differentiated liposarcomas from lipomas approached by deep learning methods
Source: Orphanet J Rare Dis. 2022 Apr 7;17:158. doi: 10.1186/s13023-022-02304-x (PMC8991509; doi:10.1186/s13023-022-02304-x)
Supplement: Supplementary file 1 — Additional file 1. Supplementary Methods, Supplementary Tables, and Supplementary Figures. [file 13023_2022_2304_MOESM1_ESM.docx]

**Supplemental Methods**

1. **Multimodality imaging acquisition**

All CT data was acquired from a simulation CT scanner (Somatom Sensation Open, Siemens Medical Solutions, Erlangen, Germany) for all patients. CT scanning was completed according the parameters as follows: 120 kVp, 250 mAs, FOV 500*500 mm, 2 mm slice. Patients were injected with 95 mL iodixanol 40~60 seconds before CT scanning. All CT images were reconstructed based on filtered back projection algorithm.

All images were acquired by 1.5-T Siemens Healthineers (Munich, Germany) MRI scanners. The median value of in-plane resolutions was 0.81 mm with a range from 0.31 to 1.88 mm. The MRI slice thickness was 5mm with reconstructed image matrix size of 512 * 512. The repetition time (TR) and echo time (TE) was 550 ms (median, range: 298-770) and 18 ms (median, range: 4-28) respectively for T1WI sequence, and 4095 (median, range: 1086-10098) and 85 ms (median, range: 30-120) respectively for T2FS sequence.

**2. Deep learning features**

2.1 Imaging preprocessing

With the masked delineation of regions of interest (ROIs) for primary tumor, we adjust the size of three consecutive axial slices with maximum tumor into 224 mm * 224 mm for the input layer of the convolutional neural networks (CNNs) models using a bounding box covering the whole tumor area. The resized images with 3 consecutive axial slices as image channels would be candidate of the CNNs for feature extraction.

2.2 CNNs architecture

A total of six base models were applied on CT, T1WI and T2FS sequences respectively for the extraction of deep learning features with representativeness, including Xception ^1^, VGG16 ^2^, VGG19 ^2^, ResNet50 ^3^, InceptionV3 ^4^, and InceptionResNetV2 ^5^. These six CNNs were commonly used and pre-trained by the large-scale and well-annotated ImageNet database ^6^. This published research released dataset containing enormous object categories and manually annotated training images, the optimization hyperparameters of which was not tuned permitting a broader generalization on other datasets. After preprocessing, prepared slices of CT and MRI images with the maximum axial area of the tumor lesion would be ready as the input of the pre-trained CNNs to generate deep learning features. The models are publicly accessed by Keras and TensorFlow open-source code (https://github.com/fchollet/deep-learning-models/).

2.3 Elimination of the last fully-connected layer

The convolutional base is connected by a fully-connected layer for the pre-trained models. We removed the last fully-connected layer, and then different CNNs reached various numbers of feature maps (2048 for ResNet50, InceptionV3 and Xception, 512 for VGG16 and VGG19, and 1536 for InceptionResNetV2) from the new output of these models.

2.4 Addition of max pooling layer and feature extraction

With the utility of a global pooling window, local data would be concentrated into a decreased dimensionality. After Step 2.3, for models with more than one-dimensional features, we got feature maps with height and width dimensions corresponding to location invariance in the input layer. After global max pooling, each feature map vector was transformed to a maximal raw value among them. During this step, the feature maps were transformed to numeric values, as representational deep learning features.

**3. Handcrafted radiomics feature**

Handcrafted radiomics features were computed from the radiologist-drawn ROIs using an open-source python package PyRadiomics (version 2.1.2) ^7^on CT, T1WI and T2FS images respectively. The online documentation of PyRadiomics package depicts the detailed formation of radiomics features (https://pyradiomics.readthedocs.io/en/latest/features.html). We set resampled voxel sizes as 1× 1 × 1 mm³ voxels for the slice thickness standardization, the bin width of image intensities as 25 HU, and voxel array shift as 1000. To allow the involvement of the whole tumor and avoid interference from the air and bone tissues, segmented voxels were resampled with the range of 50 to 400 HU. Defined radiomic image features without/with wavelet filtration were extracted to interpret tumor characteristics comprehensively. Wavelet filtration filtered original image with two pass filters, high pass filter (H) and low pass filter (L) for three directions, x, y and z respectively, which represented a total of eight different combinations of decompositions. The extracted radiomics features could be classified into three groups: (a) first-order statistics, (b) shape features, (c) second-order statistics. Most radiomics features mentioned above showed consistency with feature definitions in accordance with the IBSI guidelines (available document online ^8^).

There are differences in gray value discretization for the fixed bin size type and resampling, both of which cannot be corrected by customization settings alone and require replacement by custom functions (shown in the Pyradiomics documents). There are two features available in PyRadiomics without definitions in the IBSI, Total Energy and Standard Deviation. Entropy in Pyradiomics is defined by IBSI named Intensity Histogram Entropy. Uniformity in Pyradiomics is defined by IBSI named Intensity Histogram Uniformity. Mesh Volume in Pyradiomics is defined named Volume. Voxel Volume in Pyradiomics is defined in IBSI named Approximate Volume. Joint Energy in Pyradiomics is defined by IBSI named Angular Second Moment. Maximum Probability in Pyradiomics is defined by IBSI named Joint maximum. Sum of Squares in Pyradiomics is defined by IBSI named Joint Variance. The PyRadiomics kurtosis is not corrected, whereas IBSI kurtosis is corrected by -3, yielding 0 for normal distributions. Despite these features above, the remaining features are consistent with the IBSI definitions.

**4. Differentiation model construction**

For model building and unbiased performance evaluation on the training set, 10 iterations of five-fold nested cross validation were performed using the code published by Deist et al. built upon the “caret” package ^9, 10^. The total training set was split into five subgroups (outer folds). Each Subgroup was then split once more for five times (inner folds). Hyperparameters were optimized as part of the inner folds. The selected hyperparameters were then used for testing on the five outer folds. The total mean area under the receiver operator characteristic (ROC) curve (AUC) over all outer folds was calculated for model comparison. The hyperparameter combination with the best mean performance was used to retrain a final model on the whole training set. Model performance with the optimal hyperparameter combination was assess using cross validation.

**5. Statistical analysis**

Radiomics features were harmonized to reduce the multi-scanner disturbance resulting from different protocol parameters. Feature robustness for inter-observer reproducibility was assessed by intra-class correlation coefficients (ICCs) using “irr” R package ^11^. Discrimination ability was tested by Harrell's concordance indices (C-index) using “Hmisc” R package ^12^.

**Table.S1. Univariate and multivariate analysis on clinical variables associated with differentiation of WDLPS and lipoma in the training cohort.**

| **Characteristic** |  | **Univariate analysis** | |  | **Multivariate analysis** | |
| --- | --- | --- | --- | --- | --- | --- |
|  |  | **HR (95% CI)** | ***P* value** |  | **HR (95% CI)** | ***P* value** |
| **Gender** |  |  | 0.596 |  |  |  |
| Female |  | Reference |  |  |  |  |
| Male |  | 1.260 (0.537-2.955) |  |  |  |  |
| **Age, years** |  |  | 0.007 |  |  | 0.009 |
| ≤60 |  | Reference |  |  | Reference |  |
| >60 |  | 4.375 (1.490-12.845) |  |  | 4.299 (1.430-12.930) |  |
| **Tumor size, cm** |  |  | 0.150 |  |  |  |
| ≤10 |  | Reference |  |  |  |  |
| >10 |  | 1.922 (0.789-4.683) |  |  |  |  |
| **Tumor location** |  |  | 0.041 |  |  | 0.235 |
| Extremity |  | Reference |  |  | Reference |  |
| Trunk |  | 0.399 (0.133-1.195) |  |  | 0.419 (0.134-1.309) |  |
| Abdomen/retroperitoneal |  | 2.053 (0.706-5.965) |  |  | 1.221 (0.368-4.056) |  |
| **Tumor depth** |  |  | 0.785 |  |  |  |
| Superficial |  | Reference |  |  |  |  |
| Deep |  | 0.848 (0.260-2.766) |  |  |  |  |
| **HGB, g/L** |  |  | 0.371 |  |  |  |
| Normal |  | Reference |  |  |  |  |
| Abnormal |  | 1.641 (0.554-4.860) |  |  |  |  |
| **Platelet, 10^9/L** |  |  | 0.394 |  |  |  |
| Normal |  | Reference |  |  |  |  |
| Abnormal |  | 1.856 (0.447-7.706) |  |  |  |  |
| **WBC, 10^9/L** |  |  | 0.373 |  |  |  |
| Normal |  | Reference |  |  |  |  |
| Abnormal |  | 1.517 (0.606-3.798) |  |  |  |  |
| **ALB, g/L** |  |  | 0.324 |  |  |  |
| Normal |  | Reference |  |  |  |  |
| Abnormal |  | 1.676 (0.601-4.671) |  |  |  |  |
| **ALP, U/L** |  |  | 0.438 |  |  |  |
| Normal |  | Reference |  |  |  |  |
| Abnormal |  | 1.957 (0.359-10.676) |  |  |  |  |
| **LDH, U/L** |  |  | 0.040 |  |  | 0.050 |
| Normal |  | Reference |  |  | Reference |  |
| Abnormal |  | 5.532 (1.079-28.365) |  |  | 5.366 (0.997-28.888) |  |
| **Duration of hospitalization, day** |  |  | 0.714 |  |  |  |
| ≤14 |  | Reference |  |  |  |  |
| >14 |  | 0.846 (0.346-2.067) |  |  |  |  |

Abbreviations: WDLPS, Well-differentiated liposarcoma; HR, Hazard ratio; CI, Confidence interval; HGB, Hemoglobin; WBC, White blood cell; ALB, Serum albumin; ALP, Alkaline phosphatase; LDH, Lactate dehydrogenase.

**Table.S2. Predictive performance of multimodality (CT and MRI) deep learning and handcrafted radiomics models in classification of WDLPS and lipoma on patients in the training and validation cohorts.**

| **Model** | **Modality** | **Training cohort** | | | | | |  | **Validation cohort** | | | | | |
| --- | --- | --- | --- | --- | --- | --- | --- | --- | --- | --- | --- | --- | --- | --- |
|  |  | **AUC** | **Accuracy** | **Sensitivity** | **Specificity** | **PPV** | **NPV** |  | **AUC** | **Accuracy** | **Sensitivity** | **Specificity** | **PPV** | **NPV** |
| Xception | MRI | 0.960 (0.925-0.994) | 89.89 (80/89) | 84.21 (68.07-93.41) | 94.12 (82.77-98.47) | 91.43 (75.81-97.76) | 88.89 (76.69-95.40) |  | 0.614 (0.429-0.799) | 63.16 (24/38) | 70.00 (45.67-87.16) | 55.56 (31.35-77.60) | 63.64 (40.83-81.97) | 62.50 (35.87-83.72) |
|  | CT | 0.971 (0.942-0.999) | 91.01 (81/89) | 89.47 (74.26-96.57) | 92.16 (80.25-97.46) | 89.47 (74.26-96.57) | 92.16 (80.25-97.46) |  | 0.703 (0.527-0.878) | 65.79 (25/38) | 75.00 (50.59-90.41) | 55.56 (31.35-77.60) | 65.22 (42.82-82.81) | 66.67 (38.69-87.01) |
|  | MRI+CT | 0.973 (0.948-0.999) | 88.76 (79/89) | 97.37 (84.57-99.86) | 82.35 (68.64-91.13) | 80.43 (65.62-90.14) | 97.67 (86.20-99.88) |  | 0.739 (0.571-0.907) | 60.53 (23/38) | 80.00 (55.73-93.39) | 38.89 (18.26-63.86) | 59.26 (39.01-76.99) | 63.64 (31.61-87.63) |
|  |  |  |  |  |  |  |  |  |  |  |  |  |  |  |
| VGG16 | MRI | 0.889 (0.817-0.962) | 86.52 (77/89) | 73.68 (56.61-86.02) | 96.08 (85.41-99.32) | 93.33 (76.49-98.84) | 83.05 (70.58-91.15) |  | 0.728 (0.566-0.889) | 65.79 (25/38) | 50.00 (27.85-72.15) | 83.33 (57.74-95.59) | 76.92 (45.98-93.84) | 60.00 (38.89-78.19) |
|  | CT | 0.961 (0.927-0.996) | 89.89 (80/89) | 89.47 (74.26-96.57) | 90.20 (77.81-96.33) | 87.18 (71.77-95.18) | 92.00 (79.89-97.41) |  | 0.539 (0.352-0.726) | 52.63 (20/38) | 75.00 (50.59-90.41) | 27.78 (10.71-53.59) | 53.57 (34.21-71.99) | 50.00 (20.14-79.86) |
|  | MRI+CT | 0.947 (0.902-0.992) | 89.89 (80/89) | 84.21 (68.07-93.41) | 94.12 (82.77-98.47) | 91.43 (75.81-97.76) | 88.89 (76.69-95.40) |  | 0.703 (0.533-0.872) | 68.42 (26/38) | 60.00 (36.41-80.02) | 77.78 (51.92-92.63) | 75.00 (47.41-91.67) | 63.64 (40.83-81.97) |
|  |  |  |  |  |  |  |  |  |  |  |  |  |  |  |
| VGG19 | MRI | 0.881 (0.803-0.958) | 82.02 (73/89) | 76.32 (59.39-87.97) | 86.27 (73.13-93.85) | 80.56 (63.43-91.20) | 83.02 (69.70-91.48) |  | 0.803 (0.663-0.943) | 73.68 (28/38) | 65.00 (40.95-83.69) | 83.33 (57.74-95.59) | 81.25 (53.69-95.03) | 68.18 (45.12-85.27) |
|  | CT | 0.953 (0.914-0.992) | 88.76 (79/89) | 92.11 (77.52-97.94) | 86.27 (73.13-93.85) | 83.33 (68.04-92.49) | 93.62 (81.44-98.34) |  | 0.708 (0.533-0.884) | 71.05 (27/38) | 70.00 (45.67-87.16) | 72.22 (46.41-89.29) | 73.68 (48.58-89.88) | 68.42 (43.50-86.44) |
|  | MRI+CT | 0.921 (0.869-0.973) | 80.90 (72/89) | 97.37 (84.57-99.86) | 68.63 (53.97-50.48) | 69.81 (55.49-81.26) | 97.22 (83.80-99.85) |  | 0.786 (0.643-0.929) | 65.79 (25/38) | 90.00 (66.87-98.25) | 38.89 (18.26-63.86) | 62.07 (42.36-78.70) | 77.78 (40.19-96.05) |
|  |  |  |  |  |  |  |  |  |  |  |  |  |  |  |
| ResNet50 | MRI | 0.957 (0.922-0.992) | 87.64 (78/89) | 92.11 (77.52-97.94) | 84.31 (70.86-92.52) | 81.40 (66.08-91.08) | 93.48 (81.07-98.30) |  | 0.894 (0.797-0.992) | 76.32 (29/38) | 95.00 (73.06-99.74) | 55.56 (31.35-77.60) | 70.37 (49.66-85.50) | 90.91 (57.12-99.52) |
|  | CT | 0.989 (0.974-1.000) | 94.38 (84/89) | 89.47 (74.26-96.57) | 98.04 (88.21-99.90) | 97.14 (83.38-99.85) | 92.59 (81.26-97.60) |  | 0.894 (0.771-1.000) | 81.58 (31/38) | 75.00 (50.59-90.41) | 88.89 (63.93-98.05) | 88.24 (62.25-97.94) | 76.19 (52.45-90.88) |
|  | MRI+CT | 0.995 (0.987-1.000) | 95.51 (85/89) | 92.11 (77.52-97.94) | 98.04 (88.21-99.90) | 97.22 (83.80-99.85) | 94.34 (83.37-98.53) |  | 0.950 (0.886-1.000) | 92.11 (35/38) | 95.00 (73.06-99.74) | 88.89 (63.93-98.05) | 90.48 (68.17-98.33) | 94.12 (69.24-99.69) |
|  |  |  |  |  |  |  |  |  |  |  |  |  |  |  |
| InceptionV3 | MRI | 0.973 (0.945-1.000) | 92.13 (82/89) | 89.47 (74.26-96.57) | 94.12 (82.77-98.47) | 91.89 (76.98-97.88) | 92.31 (80.60-97.51) |  | 0.717 (0.554-0.880) | 65.79 (25/38) | 70.00 (45.67-87.16) | 61.11 (36.14-81.74) | 66.67 (43.11-84.52) | 64.71 (38.62-84.74) |
|  | CT | 0.979 (0.956-1.000) | 93.26 (83/89) | 86.84 (71.12-95.05) | 98.04 (88.21-99.90) | 97.06 (82.95-99.85) | 90.91 (79.29-96.60) |  | 0.592 (0.407-0.776) | 57.89 (22/38) | 50.00 (27.85-72.15) | 66.67 (41.15-85.64) | 62.50 (35.87-83.72) | 54.55 (32.67-74.93) |
|  | MRI+CT | 0.973 (0.945-1.000) | 92.13 (82/89) | 92.11 (77.52-97.94) | 92.16 (80.25-97.46) | 89.74 (74.84-96.66) | 94.00 (82.46-98.44) |  | 0.758 (0.604-0.913) | 60.53 (23/38) | 45.00 (23.83-67.95) | 77.78 (51.92-92.63) | 69.23 (38.88-89.64) | 56.00 (35.27-74.98) |
|  |  |  |  |  |  |  |  |  |  |  |  |  |  |  |
| InceptionResNetV2 | MRI | 0.914 (0.857-0.970) | 84.27 (75/89) | 94.74 (80.93-99.08) | 76.47 (62.18-86.75) | 75.00 (60.11-85.89) | 95.12 (82.19-99.15) |  | 0.772 (0.618-0.927) | 63.16 (24/38) | 85.00 (61.14-96.04) | 38.89 (18.26-63.86) | 60.71 (40.73-77.87) | 70.00 (35.37-91.91) |
|  | CT | 0.968 (0.937-0.999) | 89.89 (80/89) | 97.37 (84.57-99.86) | 84.31 (70.86-92.52) | 82.22 (67.42-91.49) | 97.73 (86.49-99.88) |  | 0.508 (0.318-0.699) | 52.63 (20/38) | 70.00 (45.67-87.16) | 33.33 (14.36-58.85) | 53.85 (33.75-72.86) | 50.00 (22.29-77.71) |
|  | MRI+CT | 0.945 (0.904-0.987) | 86.52 (77/89) | 89.47 (74.26-96.57) | 84.31 (70.86-92.52) | 80.95 (65.37-90.86) | 91.49 (78.73-97.24) |  | 0.731 (0.561-0.900) | 57.89 (22/38) | 80.00 (55.73-93.39) | 33.33 (14.36-58.85) | 57.14 (37.43-74.97) | 60.00 (27.37-86.31) |
|  |  |  |  |  |  |  |  |  |  |  |  |  |  |  |
| Radiomics ^a^ | MRI | 0.927 (0.875-0.978) | 84.27 (75/89) | 89.47 (74.26-96.57) | 80.39 (66.45-89.71) | 77.27 (61.78-88.01) | 91.11 (77.87-97.11) |  | 0.514 (0.325-0.703) | 55.26 (21/38) | 55.00 (32.05-76.17) | 55.56 (31.35-77.60) | 57.89 (33.97-78.88) | 52.63 (29.50-74.79) |
|  | CT | 0.990 (0.977-1.000) | 94.38 (84/89) | 94.74 (80.93-99.08) | 94.12 (82.77-98.47) | 92.31 (78.03-97.99) | 96.00 (85.14-99.30) |  | 0.603 (0.416-0.790) | 52.63 (20/38) | 45.00 (23.83-67.95) | 61.11 (36.14-81.74) | 56.25 (30.55-79.25) | 50.00 (28.80-71.20) |
|  | MRI+CT | 0.997 (0.991-1.000) | 96.63 (86/89) | 97.37 (84.57-99.86) | 96.08 (85.41-99.32) | 94.87 (81.37-99.11) | 98.00 (87.99-99.90) |  | 0.594 (0.401-0.785) | 57.89 (22/38) | 50.00 (27.85-72.15) | 66.67 (41.15-85.64) | 62.50 (35.87-83.72) | 54.55 (32.67-74.93) |

Abbreviations: WDLPS, Well-differentiated liposarcoma; MRI, Magnetic resonance imaging; CT, Computed tomography; AUC, Area under the receiver operating characteristic curve; PPV, Positive predictive value; NPV, Negative predictive value.

Note：

a: Radiomics models were constructed based on handcrafted radiomics features extracted on manual segmentation.

**Table.S3. Predictive performance of MRI-based models on different sequences in classification of WDLPS and lipoma on patients in the training and validation cohorts.**

| **Model** | **MRI sequence** | **Training cohort** | | | | | |  | **Validation cohort** | | | | | |
| --- | --- | --- | --- | --- | --- | --- | --- | --- | --- | --- | --- | --- | --- | --- |
|  |  | **AUC** | **Accuracy** | **Sensitivity** | **Specificity** | **PPV** | **NPV** |  | **AUC** | **Accuracy** | **Sensitivity** | **Specificity** | **PPV** | **NPV** |
| Xception | T1 | 0.928 (0.871-0.985) | 87.64 (78/89) | 89.47 (74.26-96.57) | 86.27 (73.13-93.85) | 82.93 (67.35-92.30) | 91.67 (79.13-97.30) |  | 0.689 (0.507-0.871) | 65.79 (25/38) | 85.00 (61.14-96.04) | 44.44 (22.40-68.65) | 62.96 (42.47-79.92) | 72.73 (39.32-92.67) |
|  | T2 | 0.954 (0.916-0.992) | 87.64 (78/89) | 92.11 (77.52-97.94) | 84.31 (70.86-92.52) | 81.40 (66.08-91.08) | 93.48 (81.07-98.30) |  | 0.472 (0.281-0.664) | 52.63 (20/38) | 45.00 (23.83-67.95) | 61.11 (36.14-81.74) | 56.25 (30.55-79.25) | 50.00 (28.80-71.20) |
|  | T1+T2 | 0.960 (0.925-0.994) | 89.89 (80/89) | 84.21 (68.07-93.41) | 94.12 (82.77-98.47) | 91.43 (75.81-97.76) | 88.89 (76.69-95.40) |  | 0.614 (0.429-0.799) | 63.16 (24/38) | 70.00 (45.67-87.16) | 55.56 (31.35-77.60) | 63.64 (40.83-81.97) | 62.50 (35.87-83.72) |
|  |  |  |  |  |  |  |  |  |  |  |  |  |  |  |
| VGG16 | T1 | 0.896 (0.830-0.962) | 80.90 (72/89) | 73.68 (56.61-86.02) | 86.27 (73.13-93.85) | 80.00 (62.54-90.94) | 81.48 (68.13-90.30) |  | 0.628 (0.447-0.808) | 55.26 (21/38) | 60.00 (36.41-80.02) | 50.00 (26.77-73.23) | 57.14 (34.44-77.41) | 52.94 (28.53-76.14) |
|  | T2 | 0.820 (0.729-0.912) | 77.53 (69/89) | 81.58 (65.11-91.68) | 74.51 (60.09-85.23) | 70.45 (54.61-82.75) | 84.44 (69.94-93.01) |  | 0.769 (0.612-0.927) | 71.05 (27/38) | 80.00 (55.73-93.39) | 61.11 (36.14-81.74) | 69.57 (46.99-85.94) | 73.33 (44.83-91.09) |
|  | T1+T2 | 0.889 (0.817-0.962) | 86.52 (77/89) | 73.68 (56.61-86.02) | 96.08 (85.41-99.32) | 93.33 (76.49-98.84) | 83.05 (70.58-91.15) |  | 0.728 (0.566-0.889) | 65.79 (25/38) | 50.00 (27.85-72.15) | 83.33 (57.74-95.59) | 76.92 (45.98-93.84) | 60.00 (38.89-78.19) |
|  |  |  |  |  |  |  |  |  |  |  |  |  |  |  |
| VGG19 | T1 | 0.885 (0.811-0.959) | 85.39 (76/89) | 73.68 (56.61-86.02) | 94.12 (82.77-98.47) | 90.32 (73.10-97.47) | 82.76 (70.12-90.99) |  | 0.783 (0.626-0.941) | 73.68 (28/38) | 70.00 (45.67-87.16) | 77.78 (51.92-92.63) | 77.78 (51.92-92.63) | 70.00 (45.67-87.16) |
|  | T2 | 0.849 (0.770-0.929) | 77.53 (69/89) | 78.95 (62.22-89.86) | 76.47 (62.18-86.75) | 71.43 (55.20-83.77) | 82.98 (68.65-91.86) |  | 0.767 (0.611-0.922) | 76.32 (29/38) | 75.00 (50.59-90.41) | 77.78 (51.92-92.63) | 78.95 (53.90-93.03) | 73.68 (48.58-89.88) |
|  | T1+T2 | 0.881 (0.803-0.958) | 82.02 (73/89) | 76.32 (59.39-87.97) | 86.27 (73.13-93.85) | 80.56 (63.43-91.20) | 83.02 (69.70-91.48) |  | 0.803 (0.663-0.943) | 73.68 (28/38) | 65.00 (40.95-83.69) | 83.33 (57.74-95.59) | 81.25 (53.69-95.03) | 68.18 (45.12-85.27) |
|  |  |  |  |  |  |  |  |  |  |  |  |  |  |  |
| ResNet50 | T1 | 0.973 (0.946-1.000) | 92.13 (82/89) | 86.84 (71.12-95.05) | 96.08 (85.41-99.32) | 94.29 (79.48-99.00) | 90.74 (78.94-96.54) |  | 0.947 (0.878-1.000) | 73.68 (28/38) | 95.00 (73.06-99.74) | 50.00 (26.77-73.23) | 67.86 (47.57-83.42) | 90.00 (54.12-99.48) |
|  | T2 | 0.861 (0.786-0.937) | 80.90 (72/89) | 73.68 (56.61-86.02) | 86.27 (73.13-93.85) | 80.00 (62.54-90.94) | 81.48 (68.13-90.30) |  | 0.669 (0.491-0.848) | 71.05 (27/38) | 60.00 (36.41-80.02) | 83.33 (57.74-95.59) | 80.00 (51.37-94.69) | 65.22 (42.82-82.81) |
|  | T1+T2 | 0.957 (0.922-0.992) | 87.64 (78/89) | 92.11 (77.52-97.94) | 84.31 (70.86-92.52) | 81.40 (66.08-91.08) | 93.48 (81.07-98.30) |  | 0.894 (0.797-0.992) | 76.32 (29/38) | 95.00 (73.06-99.74) | 55.56 (31.35-77.60) | 70.37 (49.66-85.50) | 90.91 (57.12-99.52) |
|  |  |  |  |  |  |  |  |  |  |  |  |  |  |  |
| InceptionV3 | T1 | 0.915 (0.858-0.972) | 83.15 (74/89) | 76.32 (59.39-87.97) | 88.24 (75.44-95.13) | 82.86 (65.70-92.83) | 83.33 (70.21-91.64) |  | 0.669 (0.494-0.845) | 63.16 (24/38) | 60.00 (36.41-80.02) | 66.67 (41.15-85.64) | 66.67 (41.15-85.64) | 60.00 (36.41-80.02) |
|  | T2 | 0.957 (0.922-0.992) | 86.52 (77/89) | 89.47 (74.26-96.57) | 84.31 (70.86-92.52) | 80.95 (65.37-90.86) | 91.49 (78.73-97.24) |  | 0.650 (0.471-0.829) | 63.16 (24/38) | 55.00 (32.05-76.17) | 72.22 (46.41-89.29) | 68.75 (41.48-87.87) | 59.09 (36.68-78.52) |
|  | T1+T2 | 0.973 (0.945-1.000) | 92.13 (82/89) | 89.47 (74.26-96.57) | 94.12 (82.77-98.47) | 91.89 (76.98-97.88) | 92.31 (80.60-97.51) |  | 0.717 (0.554-0.880) | 65.79 (25/38) | 70.00 (45.67-87.16) | 61.11 (36.14-81.74) | 66.67 (43.11-84.52) | 64.71 (38.62-84.74) |
|  |  |  |  |  |  |  |  |  |  |  |  |  |  |  |
| InceptionResNetV2 | T1 | 0.897 (0.833-0.960) | 83.15 (74/89) | 78.95 (62.22-89.86) | 86.27 (73.13-93.85) | 81.08 (64.29-91.44) | 84.62 (71.37-92.66) |  | 0.772 (0.620-0.924) | 73.68 (28/38) | 85.00 (61.14-96.04) | 61.11 (36.14-81.74) | 70.83 (48.75-86.56) | 78.57 (48.82-94.29) |
|  | T2 | 0.843 (0.755-0.931) | 78.65 (70/89) | 81.58 (65.11-91.68) | 76.47 (62.18-86.75) | 72.09 (56.10-84.17) | 84.78 (70.52-93.16) |  | 0.694 (0.525-0.864) | 55.26 (21/38) | 50.00 (27.85-72.15) | 61.11 (36.14-81.74) | 58.82 (33.45-80.57) | 52.38 (30.34-73.61) |
|  | T1+T2 | 0.914 (0.857-0.970) | 84.27 (75/89) | 94.74 (80.93-99.08) | 76.47 (62.18-86.75) | 75.00 (60.11-85.89) | 95.12 (82.19-99.15) |  | 0.772 (0.618-0.927) | 63.16 (24/38) | 85.00 (61.14-96.04) | 38.89 (18.26-63.86) | 60.71 (40.73-77.87) | 70.00 (35.37-91.91) |
|  |  |  |  |  |  |  |  |  |  |  |  |  |  |  |
| Radiomics ^a^ | T1 | 0.929 (0.875-0.983) | 89.89 (80/89) | 81.58 (65.11-91.68) | 96.08 (85.41-99.32) | 93.94 (78.38-98.94) | 87.50 (75.31-94.41) |  | 0.531 (0.341-0.720) | 47.37 (18/38) | 35.00 (16.31-59.05) | 61.11 (36.14-81.74) | 50.00 (24.04-75.96) | 45.83 (26.17-66.76) |
|  | T2 | 0.895 (0.823-0.966) | 80.90 (72/89) | 86.84 (71.12-95.05) | 76.47 (62.18-86.75) | 73.33 (57.79-84.90) | 88.64 (74.65-95.74) |  | 0.586 (0.400-0.772) | 60.53 (23/38) | 65.00 (40.95-83.69) | 55.56 (31.35-77.60) | 61.90 (38.69-81.05) | 58.82 (33.45-80.57) |
|  | T1+T2 | 0.927 (0.875-0.978) | 84.27 (75/89) | 89.47 (74.26-96.57) | 80.39 (66.45-89.71) | 77.27 (61.78-88.01) | 91.11 (77.87-97.11) |  | 0.514 (0.325-0.703) | 55.26 (21/38) | 55.00 (32.05-76.17) | 55.56 (31.35-77.60) | 57.89 (33.97-78.88) | 52.63 (29.50-74.79) |

Abbreviations: WDLPS, Well-differentiated liposarcoma; MRI, Magnetic resonance imaging; AUC, Area under the receiver operating characteristic curve; PPV, Positive predictive value; NPV, Negative predictive value.

Note：

a: Radiomics models were constructed based on handcrafted radiomics features extracted on manual segmentation.

**Table.S4. Predictive performance of deep learning-based models on different imaging examination in classification of WDLPS and lipoma during cross-validation of model construction.**

| **Model** | | **Modality** | **Cross validation** | | | | | |
| --- | --- | --- | --- | --- | --- | --- | --- | --- |
|  |  |  | **AUC** | **Accuracy** | **Sensitivity** | **Specificity** | **PPV** | **NPV** |
| Xception | | T1 | 0.867-0.928 | 77.78-87.64 | 76.47-89.47 | 78.95-86.27 | 76.47-82.93 | 78.95-91.67 |
|  |  | T2 | 0.938-0.954 | 86.11-87.64 | 88.24-92.11 | 84.21-84.31 | 81.40-83.33 | 88.89-93.48 |
|  |  | MRI | 0.913-0.960 | 80.56-89.89 | 76.47-84.21 | 84.21-94.12 | 81.25-91.43 | 80.00-88.89 |
|  |  | CT | 0.893-0.971 | 80.56-91.01 | 88.24-89.47 | 73.68-92.16 | 75.00-89.47 | 87.50-92.16 |
|  |  | CT+MRI | 0.947-0.973 | 86.11-88.76 | 94.12-97.37 | 78.95-82.35 | 80.00-80.43 | 93.75-97.67 |
|  | |  |  |  |  |  |  |  |
| VGG16 | | T1 | 0.805-0.896 | 72.22-80.90 | 58.82-73.68 | 84.21-86.27 | 76.92-80.00 | 69.57-81.48 |
|  |  | T2 | 0.749-0.820 | 72.22-77.53 | 76.47-81.58 | 68.42-74.51 | 68.42-70.45 | 76.47-84.44 |
|  |  | MRI | 0.740-0.889 | 72.22-86.52 | 52.94-73.68 | 89.47-96.08 | 81.82-93.33 | 68.00-83.05 |
|  |  | CT | 0.950-0.961 | 88.89-89.89 | 88.24-89.47 | 89.47-90.20 | 87.18-88.24 | 89.47-92.00 |
|  |  | CT+MRI | 0.898-0.947 | 86.11-89.89 | 82.35-84.21 | 89.47-94.12 | 87.50-91.43 | 85.00-88.89 |
|  | |  |  |  |  |  |  |  |
| VGG19 | | T1 | 0.836-0.885 | 75.00-85.39 | 58.82-73.68 | 89.47-94.12 | 83.33-90.32 | 70.83-82.76 |
|  |  | T2 | 0.824-0.849 | 75.00-77.53 | 76.47-78.95 | 73.68-76.47 | 71.43-72.22 | 77.78-82.98 |
|  |  | MRI | 0.783-0.881 | 72.22-82.02 | 58.82-76.32 | 84.21-86.27 | 76.92-80.56 | 69.57-83.02 |
|  |  | CT | 0.950-0.953 | 87.89-88.76 | 92.11-100.00 | 78.95-86.27 | 80.95-83.33 | 93.62-100.00 |
|  |  | CT+MRI | 0.861-0.921 | 75.00-80.90 | 97.37-100.00 | 52.63-68.63 | 65.38-69.81 | 97.22-100.00 |
|  | |  |  |  |  |  |  |  |
| InceptionV3 | | T1 | 0.908-0.915 | 80.33-83.15 | 76.32-82.35 | 84.21-88.24 | 82.35-82.86 | 83.33-84.21 |
|  |  | T2 | 0.913-0.957 | 80.56-86.52 | 88.24-89.47 | 73.68-84.31 | 75.00-80.95 | 87.50-91.49 |
|  |  | MRI | 0.945-0.973 | 91.67-92.13 | 80.26-89.47 | 89.47-94.12 | 88.89-91.89 | 92.31-94.44 |
|  |  | CT | 0.967-0.979 | 90.22-93.26 | 84.12-86.84 | 90.00-98.04 | 90.00-97.06 | 90.91-95.00 |
|  |  | CT+MRI | 0.947-0.973 | 88.89-92.13 | 92.11-94.12 | 84.21-92.16 | 84.21-89.74 | 94.00-94.12 |
|  | |  |  |  |  |  |  |  |
| InceptionResNetV2 | | T1 | 0.864-0.897 | 80.33-83.15 | 70.59-78.95 | 86.27-94.74 | 81.08-92.31 | 78.26-84.62 |
|  |  | T2 | 0.802-0.843 | 77.78-78.65 | 76.47-81.58 | 76.47-78.95 | 72.09-76.47 | 78.95-84.78 |
|  |  | MRI | 0.870-0.914 | 83.33-84.27 | 88.24-94.74 | 76.47-78.95 | 75.00-78.95 | 88.24-95.12 |
|  |  | CT | 0.941-0.968 | 86.11-89.89 | 97.37-100.00 | 73.68-84.31 | 77.27-82.22 | 97.73-100.00 |
|  |  | CT+MRI | 0.913-0.945 | 83.33-86.52 | 82.35-89.47 | 84.21-84.31 | 80.95-82.35 | 84.21-91.49 |
|  | |  |  |  |  |  |  |  |
| ResNet50 | Res2b | T1 | 0.737-0.825 | 75.00-79.78 | 58.82-68.42 | 88.24-89.47 | 81.25-83.33 | 70.83-78.95 |
|  |  | T2 | 0.710-0.731 | 71.78-74.16 | 50.00-64.71 | 89.47-92.16 | 82.61-84.62 | 71.21-73.91 |
|  |  | MRI | 0.703-0.806 | 75.00-78.65 | 64.71-73.68 | 82.35-84.21 | 75.68-78.57 | 72.73-80.77 |
|  |  | CT | 0.892-0.922 | 83.33-86.52 | 94.12-94.74 | 73.68-80.39 | 76.19-78.26 | 93.33-95.35 |
|  |  | CT+MRI | 0.718-0.830 | 69.44-78.65 | 58.82-71.05 | 78.95-84.31 | 71.43-77.14 | 68.18-79.63 |
|  |  |  |  |  |  |  |  |  |
|  | Res3d | T1 | 0.793-0.846 | 72.22-76.40 | 76.47-84.21 | 68.42-70.59 | 68.09-68.42 | 76.47-85.71 |
|  |  | T2 | 0.697-0.808 | 58.33-71.91 | 52.94-60.53 | 63.16-80.39 | 56.25-69.70 | 60.00-73.21 |
|  |  | MRI | 0.805-0.850 | 75.00-77.53 | 76.47-86.84 | 70.59-73.68 | 68.75-72.22 | 77.78-87.80 |
|  |  | CT | 0.935-0.938 | 81.67-86.52 | 90.00-92.11 | 82.35-84.21 | 79.55-85.00 | 93.33-100.00 |
|  |  | CT+MRI | 0.808-0.872 | 75.00-80.90 | 64.71-76.32 | 84.21-84.31 | 78.38-78.57 | 72.73-82.69 |
|  |  |  |  |  |  |  |  |  |
|  | Res4f | T1 | 0.862-0.879 | 73.33-78.65 | 88.24-89.47 | 70.59-78.95 | 69.39-78.95 | 88.24-90.00 |
|  |  | T2 | 0.864-0.931 | 80.56-84.27 | 82.35-86.84 | 78.95-82.35 | 77.78-78.57 | 83.33-89.36 |
|  |  | MRI | 0.847-0.920 | 72.22-84.27 | 70.59-81.58 | 73.68-86.27 | 70.59-81.58 | 73.68-86.27 |
|  |  | CT | 0.972-0.990 | 88.89-94.38 | 82.35-89.47 | 94.74-98.04 | 93.33-97.14 | 85.71-92.59 |
|  |  | CT+MRI | 0.938-0.957 | 86.11-91.01 | 76.47-86.84 | 94.12-94.74 | 91.67-92.86 | 81.82-90.57 |
|  |  |  |  |  |  |  |  |  |
|  | Res5c | T1 | 0.944-0.973 | 86.11-92.13 | 82.35-86.84 | 89.47-96.08 | 87.50-94.29 | 85.00-90.74 |
|  |  | T2 | 0.827-0.861 | 77.78-80.90 | 73.68-76.47 | 78.95-86.27 | 76.47-80.00 | 78.95-81.48 |
|  |  | MRI | 0.923-0.957 | 80.56-87.64 | 92.11-94.12 | 68.42-84.31 | 72.73-81.40 | 92.86-93.48 |
|  |  | CT | 0.988-0.989 | 91.67-94.38 | 89.47-94.12 | 89.47-98.04 | 88.89-97.14 | 92.59-94.44 |
|  |  | CT+MRI | 0.975-0.995 | 88.89-95.51 | 88.24-92.11 | 89.47-98.04 | 88.24-97.22 | 89.47-94.34 |
|  |  |  |  |  |  |  |  |  |
|  | FC1000 | T1 | 0.824-0.836 | 74.78-75.28 | 76.47-94.74 | 60.78-78.95 | 64.29-76.47 | 78.95-93.94 |
|  |  | T2 | 0.809-0.822 | 72.00-75.28 | 68.42-88.24 | 63.16-80.39 | 68.18-72.22 | 77.36-85.71 |
|  |  | MRI | 0.809-0.822 | 72.00-75.28 | 68.42-88.24 | 63.16-80.39 | 68.18-72.22 | 77.36-85.71 |
|  |  | CT | 0.916-0.949 | 77.78-89.89 | 97.37-100.00 | 57.89-84.31 | 68.00-82.22 | 97.73-100.00 |
|  |  | CT+MRI | 0.925-0.956 | 91.44-94.38 | 97.37-100.00 | 89.47-92.16 | 89.47-90.24 | 97.92-100.00 |

Abbreviations: WDLPS, Well-differentiated liposarcoma; MRI, Magnetic resonance imaging; CT, Computed tomography; AUC, Area under the receiver operating characteristic curve; PPV, Positive predictive value; NPV, Negative predictive value.

**Table.S5. Number of features selected and used in construction of deep learning-based models.**

| **Feature extractor** |  | **Modality** | **Number of selected features** |
| --- | --- | --- | --- |
| Xception |  | T1 | 3 |
|  |  | T2 | 7 |
|  |  | MRI | 4 |
|  |  | CT | 6 |
|  |  |  |  |
| VGG16 |  | T1 | 5 |
|  |  | T2 | 3 |
|  |  | MRI | 4 |
|  |  | CT | 8 |
|  |  |  |  |
| VGG19 |  | T1 | 5 |
|  |  | T2 | 4 |
|  |  | MRI | 5 |
|  |  | CT | 6 |
|  |  |  |  |
| ResNet50 |  | T1 | 3 |
|  |  | T2 | 4 |
|  |  | MRI | 3 |
|  |  | CT | 2 |
|  |  |  |  |
| InceptionV3 |  | T1 | 4 |
|  |  | T2 | 5 |
|  |  | MRI | 7 |
|  |  | CT | 7 |
|  |  |  |  |
| InceptionResNetV2 |  | T1 | 3 |
|  |  | T2 | 4 |
|  |  | MRI | 4 |
|  |  | CT | 8 |

Abbreviations: MRI, Magnetic resonance imaging; CT, Computed tomography.

**Table.S6. Predictive performances of deep learning-based models constructed by features extracted from different layers of ResNet50 algorithm in training and validation cohorts.**

| **Layer** | **Modality** | **Training cohort** | | | | | |  | **Validation cohort** | | | | | |
| --- | --- | --- | --- | --- | --- | --- | --- | --- | --- | --- | --- | --- | --- | --- |
|  |  | **AUC** | **Accuracy** | **Sensitivity** | **Specificity** | **PPV** | **NPV** |  | **AUC** | **Accuracy** | **Sensitivity** | **Specificity** | **PPV** | **NPV** |
| Res2b | T1 | 0.825 (0.732-0.918) | 79.78 (71/89) | 68.42 (51.21-81.96) | 88.24 (75.44-95.13) | 81.25 (62.96-92.14) | 78.95 (65.75-88.20) |  | 0.750 (0.589-0.911) | 71.05 (27/38) | 65.00 (40.95-83.69) | 77.78 (51.92-92.63) | 76.47 (49.76-92.18) | 66.67 (43.11-84.52) |
|  | T2 | 0.731 (0.619-0.842) | 74.16 (66/89) | 50.00 (33.66-66.34) | 92.16 (80.25-97.46) | 82.61 (60.45-94.28) | 71.21 (58.57-81.36) |  | 0.650 (0.470-0.830) | 68.42 (26/38) | 55.00 (32.05-76.17) | 83.33 (57.74-95.59) | 78.57 (48.82-94.29) | 62.50 (40.76-80.45) |
|  | MRI | 0.806 (0.708-0.904) | 78.65 (70/89) | 73.68 (56.61-86.02) | 82.35 (68.64-91.13) | 75.68 (58.45-87.63) | 80.77 (67.03-89.92) |  | 0.689 (0.517-0.861) | 65.79 (25/38) | 85.00 (61.14-96.04) | 44.44 (22.40-68.65) | 62.96 (42.47-79.92) | 72.73 (39.32-92.67) |
|  | CT | 0.922 (0.864-0.981) | 86.52 (77/89) | 94.74 (80.93-99.08) | 80.39 (66.45-89.71) | 78.26 (63.24-88.55) | 95.35 (82.94-99.19) |  | 0.792 (0.643-0.941) | 73.68 (28/38) | 80.00 (55.73-93.39) | 66.67 (41.15-85.64) | 72.73 (49.56-88.39) | 75.00 (47.41-91.67) |
|  | CT+MRI | 0.830 (0.742-0.917) | 78.65 (70/89) | 71.05 (53.89-84.02) | 84.31 (70.86-92.52) | 77.14 (59.45-88.96) | 79.63 (59.45-88.96) |  | 0.703 (0.536-0.869) | 57.89 (22/38) | 60.00 (36.41-80.02) | 55.56 (31.35-77.60) | 60.00 (36.41-80.02) | 55.56 (31.35-77.60) |
|  |  |  |  |  |  |  |  |  |  |  |  |  |  |  |
| Res3d | T1 | 0.846 (0.762-0.931) | 76.40 (68/89) | 84.21 (68.07-93.41) | 70.59 (55.98-82.09) | 68.09 (52.75-80.48) | 85.71 (70.77-94.06) |  | 0.783 (0.639-0.928) | 63.16 (24/38) | 80.00 (55.73-93.39) | 44.44 (22.40-68.65) | 61.54 (40.73-79.09) | 66.67 (35.44-88.73) |
|  | T2 | 0.808 (0.721-0.895) | 71.91 (64/89) | 60.53 (43.45-75.51) | 80.39 (66.45-89.71) | 69.70 (51.13-83.79) | 73.21 (59.46-83.77) |  | 0.703 (0.534-0.872) | 65.79 (25/38) | 70.00 (45.67-87.16) | 61.11 (36.14-81.74) | 66.67 (43.11-84.52) | 64.71 (38.62-84.74) |
|  | MRI | 0.850 (0.764-0.935) | 77.53 (69/89) | 86.84 (71.12-95.05) | 70.59 (55.98-82.09) | 68.75 (53.60-80.91) | 87.80 (73.00-95.42) |  | 0.786 (0.641-0.931) | 68.42 (26/38) | 85.00 (61.14-96.04) | 50.00 (26.77-73.23) | 65.38 (44.37-82.06) | 75.00 (42.84-93.31) |
|  | CT | 0.938 (0.887-0.989) | 86.52 (77/89) | 92.11 (77.52-97.94) | 82.35 (68.64-91.13) | 79.55 (64.25-89.67) | 93.33 (80.69-98.26) |  | 0.789 (0.644-0.933) | 71.05 (27/38) | 65.00 (40.95-83.69) | 77.78 (51.92-92.63) | 76.47 (49.76-92.18) | 66.67 (43.11-84.52) |
|  | CT+MRI | 0.872 (0.798-0.945) | 80.90 (72/89) | 76.32 (59.39-87.97) | 84.31 (70.86-92.52) | 78.38 (61.34-89.58) | 82.69 (69.18-91.31) |  | 0.719 (0.554-0.885) | 63.16 (24/38) | 70.00 (45.67-87.16) | 55.56 (31.35-77.60) | 63.64 (40.83-81.97) | 62.50 (35.87-83.72) |
|  |  |  |  |  |  |  |  |  |  |  |  |  |  |  |
| Res4f | T1 | 0.879 (0.812-0.947) | 78.65 (70/89) | 89.47 (74.26-96.57) | 70.59 (55.98-82.09) | 69.39 (54.42-81.32) | 90.00 (75.40-96.75) |  | 0.761 (0.608-0.915) | 65.79 (25/38) | 80.00 (55.73-93.39) | 50.00 (26.77-73.23) | 64.00 (42.62-81.29) | 69.23 (38.88-89.64) |
|  | T2 | 0.931 (0.883-0.979) | 84.27 (75/89) | 86.84 (71.12-95.05) | 82.35 (68.64-91.13) | 78.57 (62.76-89.16) | 89.36 (76.11-96.02) |  | 0.550 (0.357-0.743) | 55.26 (21/38) | 50.00 (27.85-72.15) | 61.11 (36.14-81.74) | 58.82 (33.45-80.57) | 52.38 (30.34-73.61) |
|  | MRI | 0.920 (0.867-0.973) | 84.27 (75/89) | 81.58 (65.11-91.68) | 86.27 (73.13-93.85) | 81.58 (65.11-91.68) | 86.27 (73.13-93.85) |  | 0.669 (0.491-0.848) | 60.53 (23/38) | 60.00 (36.41-80.02) | 61.11 (36.14-81.74) | 63.16 (38.63-82.77) | 57.89 (33.97-78.88) |
|  | CT | 0.990 (0.976-1.000) | 94.38 (84/89) | 89.47 (74.26-96.57) | 98.04 (88.21-99.90) | 97.14 (83.38-99.85) | 92.59 (81.26-97.60) |  | 0.764 (0.612-0.916) | 63.16 (24/38) | 50.00 (27.85-72.15) | 77.78 (51.92-92.63) | 71.43 (42.00-90.42) | 58.33 (36.94-77.20) |
|  | CT+MRI | 0.957 (0.919-0.994) | 91.01 (81/89) | 86.84 (71.12-95.05) | 94.12 (82.77-98.47) | 91.67 (76.41-97.82) | 90.57 (78.58-96.47) |  | 0.681 (0.510-0.851) | 63.16 (24/38) | 55.00 (32.05-76.17) | 72.22 (46.41-89.29) | 68.75 (41.48-87.87) | 59.09 (36.68-78.52) |
|  |  |  |  |  |  |  |  |  |  |  |  |  |  |  |
| **Res5c** | **T1** | **0.973 (0.946-1.000)** | **92.13 (82/89)** | **86.84 (71.12-95.05)** | **96.08 (85.41-99.32)** | **94.29 (79.48-99.00)** | **90.74 (78.94-96.54)** |  | **0.947 (0.878-1.000)** | **73.68 (28/38)** | **95.00 (73.06-99.74)** | **50.00 (26.77-73.23)** | **67.86 (47.57-83.42)** | **90.00 (54.12-99.48)** |
|  | **T2** | **0.861 (0.786-0.937)** | **80.90 (72/89)** | **73.68 (56.61-86.02)** | **86.27 (73.13-93.85)** | **80.00 (62.54-90.94)** | **81.48 (68.13-90.30)** |  | **0.669 (0.491-0.848)** | **71.05 (27/38)** | **60.00 (36.41-80.02)** | **83.33 (57.74-95.59)** | **80.00 (51.37-94.69)** | **65.22 (42.82-82.81)** |
|  | **MRI** | **0.957 (0.922-0.992)** | **87.64 (78/89)** | **92.11 (77.52-97.94)** | **84.31 (70.86-92.52)** | **81.40 (66.08-91.08)** | **93.48 (81.07-98.30)** |  | **0.894 (0.797-0.992)** | **76.32 (29/38)** | **95.00 (73.06-99.74)** | **55.56 (31.35-77.60)** | **70.37 (49.66-85.50)** | **90.91 (57.12-99.52)** |
|  | **CT** | **0.989 (0.974-1.000)** | **94.38 (84/89)** | **89.47 (74.26-96.57)** | **98.04 (88.21-99.90)** | **97.14 (83.38-99.85)** | **92.59 (81.26-97.60)** |  | **0.894 (0.771-1.000)** | **81.58 (31/38)** | **75.00 (50.59-90.41)** | **88.89 (63.93-98.05)** | **88.24 (62.25-97.94)** | **76.19 (52.45-90.88)** |
|  | **CT+MRI** | **0.995 (0.987-1.000)** | **95.51 (85/89)** | **92.11 (77.52-97.94)** | **98.04 (88.21-99.90)** | **97.22 (83.80-99.85)** | **94.34 (83.37-98.53)** |  | **0.950 (0.886-1.000)** | **92.11 (35/38)** | **95.00 (73.06-99.74)** | **88.89 (63.93-98.05)** | **90.48 (68.17-98.33)** | **94.12 (69.24-99.69)** |
|  |  |  |  |  |  |  |  |  |  |  |  |  |  |  |
| FC1000 | T1 | 0.836 (0.754-0.918) | 75.28 (67/89) | 94.74 (80.93-99.08) | 60.78 (46.12-73.83) | 64.29 (50.30-76.31) | 93.94 (78.38-98.94) |  | 0.714 (0.547-0.881) | 60.53 (23/38) | 80.00 (55.73-93.39) | 38.89 (18.26-63.86) | 59.26 (39.01-76.99) | 63.64 (31.61-87.63) |
|  | T2 | 0.822 (0.736-0.908) | 75.28 (67/89) | 68.42 (51.21-81.96) | 80.39 (66.45-89.71) | 72.22 (54.57-85.21) | 77.36 (63.45-87.27) |  | 0.569 (0.383-0.756) | 47.37 (18/38) | 45.00 (23.83-67.95) | 50.00 (26.77-73.23) | 50.00 (26.77-73.23) | 45.00 (23.83-67.95) |
|  | MRI | 0.822 (0.736-0.908) | 75.28 (67/89) | 68.42 (51.21-81.96) | 80.39 (66.45-89.71) | 72.22 (54.57-85.21) | 77.36 (63.45-87.27) |  | 0.569 (0.383-0.756) | 47.37 (18/38) | 45.00 (23.83-67.95) | 50.00 (26.77-73.23) | 50.00 (26.77-73.23) | 45.00 (23.83-67.95) |
|  | CT | 0.949 (0.905-0.994) | 89.89 (80/89) | 97.37 (84.57-99.86) | 84.31 (70.86-92.52) | 82.22 (67.42-91.49) | 97.73 (86.49-99.88) |  | 0.578 (0.382-0.774) | 60.53 (23/38) | 70.00 (45.67-87.16) | 50.00 (26.77-73.23) | 60.87 (38.78-79.53) | 60.00 (32.89-82.54) |
|  | CT+MRI | 0.956 (0.912-1.000) | 94.38 (84/89) | 97.37 (84.57-99.86) | 92.16 (80.25-97.46) | 90.24 (75.94-96.83) | 97.92 (87.53-99.89) |  | 0.864 (0.722-1.000) | 89.47 (34/38) | 100.00 (79.95-100.00) | 77.78 (51.92-92.63) | 83.33 (61.81-94.52) | 100.00 (73.24-100.00) |

Abbreviations: WDLPS, Well-differentiated liposarcoma; MRI, Magnetic resonance imaging; CT, Computed tomography; AUC, Area under the receiver operating characteristic curve; PPV, Positive predictive value; NPV, Negative predictive value.

**Table.S7. Handcrafted radiomics features for differentiation of WDLPS and lipoma.**

| **Modality** | **Index** | **Filter ^a^** | **Feature class** | **Feature** |
| --- | --- | --- | --- | --- |
| T1 | 1 | T1_Wavelet ^c^ (HLH) | GLCM | Inverse Difference Moment Normalized |
|  | 2 | T1_wavelet (LLL) | GLCM | Cluster Shade |
|  | 3 | T1_wavelet (LLL) | GLSZM | Gray Level Non Uniformity Normalized |
|  | 4 | T1_Wavelet (HLH) | First order ^d^ | Skewness |
|  | 5 | T1_Wavelet (HLL) | First order | Kurtosis |
|  | 6 | T1_Wavelet (LHH) | First order | Small Dependence Low Gray Level Emphasis |
|  | 7 | T1_Original ^b^ | GLCM | Informational Measure of Correlation 2 |
|  | 8 | T1_Wavelet (LHH) | GLCM | Maximal Correlation Coefficient |
|  |  |  |  |  |
| T2 | 1 | T2_Wavelet (LHH) | GLCM | Maximal Correlation Coefficient |
|  | 2 | T2_Wavelet (LHL) | First order | Skewness |
|  | 3 | T2_Original | GLSZM | Size Zone Non Uniformity Normalized |
|  | 4 | T2_Wavelet (LLH) | GLCM | Correlation |
|  | 5 | T2_Original | GLCM | Maximal Correlation Coefficient |
|  |  |  |  |  |
| MRI | 1 | T1_Wavelet (LLH) | GLCM | Informational Measure of Correlation 1 |
|  | 2 | T2_Wavelet (LHH) | GLCM | Maximal Correlation Coefficient |
|  | 3 | T2_Wavelet (LLL) | GLDM | Dependence Variance |
|  | 4 | T2_Wavelet (LLH) | GLCM | Correlation |
|  | 5 | T2_Original | GLDM | Small Dependence Emphasis |
|  | 6 | T1_Wavelet (LHH) | First order | Small Dependence Low Gray Level Emphasis |
|  | 7 | T1_Original | Shape | Minor Axis Length |
|  |  |  |  |  |
| CT | 1 | CT_Original | Shape | Elongation |
|  | 2 | CT_Wavelet (LLH) | GLCM | Inverse Variance |

Abbreviations: WDLPS, Well differentiated liposarcoma; MRI, Magnetic resonance imaging; CT, Computed tomography; GLCM, Gray Level Co-occurrence Matrix Features; GLSZM, Gray Level Size Zone Matrix Features; GLDM, .Gray Level Dependence Matrix Features.

a: HLH, HLL, HHL, HHH and LLL, representative of high pass or low pass filter on the X, Y, Z three dimensions (H, high pass filter; L, low pass filter);

b: Original, original images without any filter used;

c: Wavelet, wavelet filtrated image;

d: First order, first order statistics.

**Figure.S1. Evaluation of predictive performance of the clinical model in differentiation of WDLPS and lipoma on patients in the training and validation cohorts.**


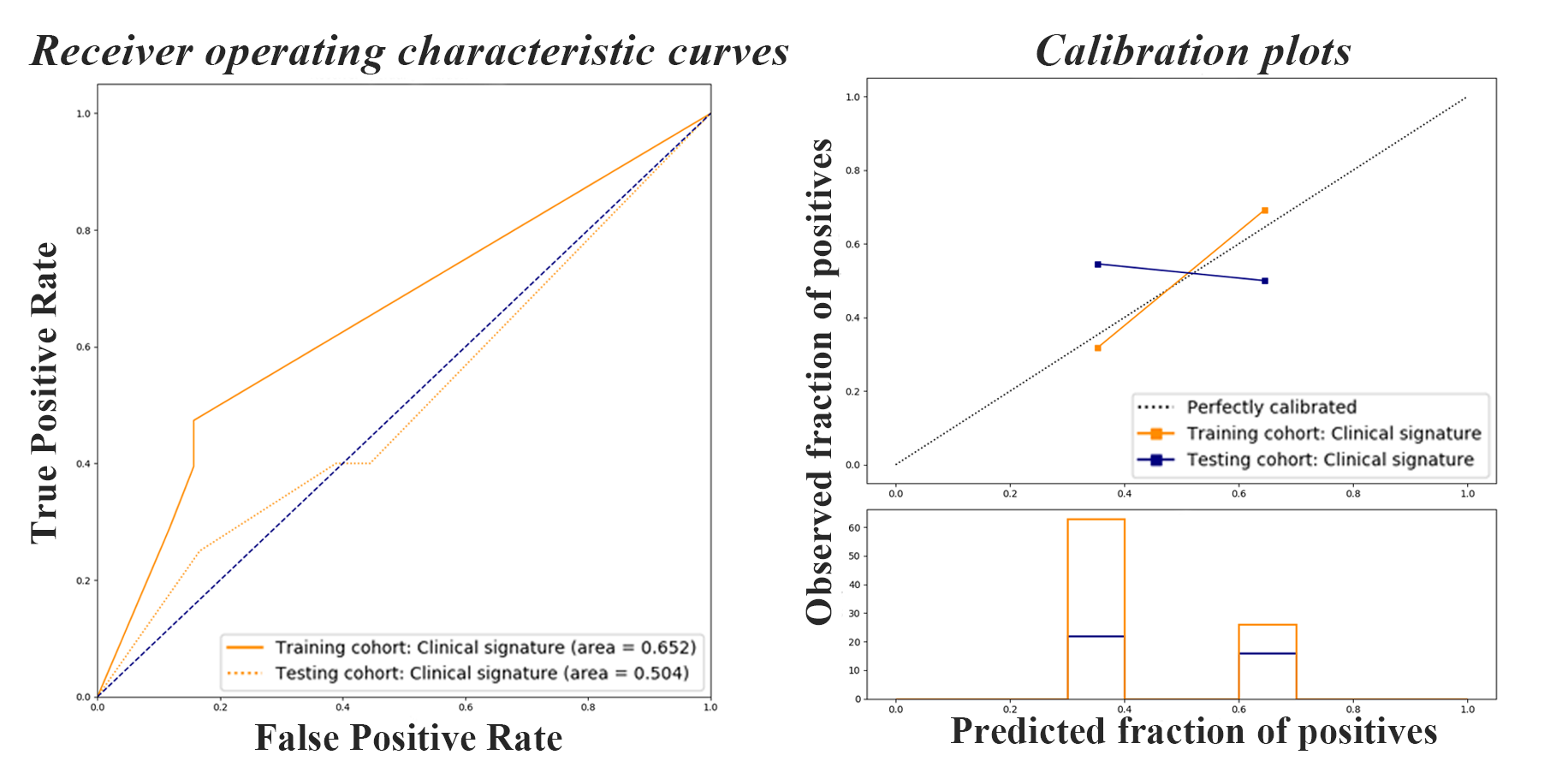


Evaluation of predictive performances for clinical models using clinical variables, age at diagnosis and LDH level, selected by multivariate analysis with p value less than 0.05 in the training cohort via ROC analysis, precision-recall plots, and calibration analysis.

Abbreviations: WDLPS, Well-differentiated liposarcoma; ROC, Receiver operating characteristic.

**Figure.S2. Evaluation of predictive performance of deep learning-based models on T1- or T2-derived MRI sequence features in classification of WDLPS and lipoma on patients in the training and validation cohorts.**


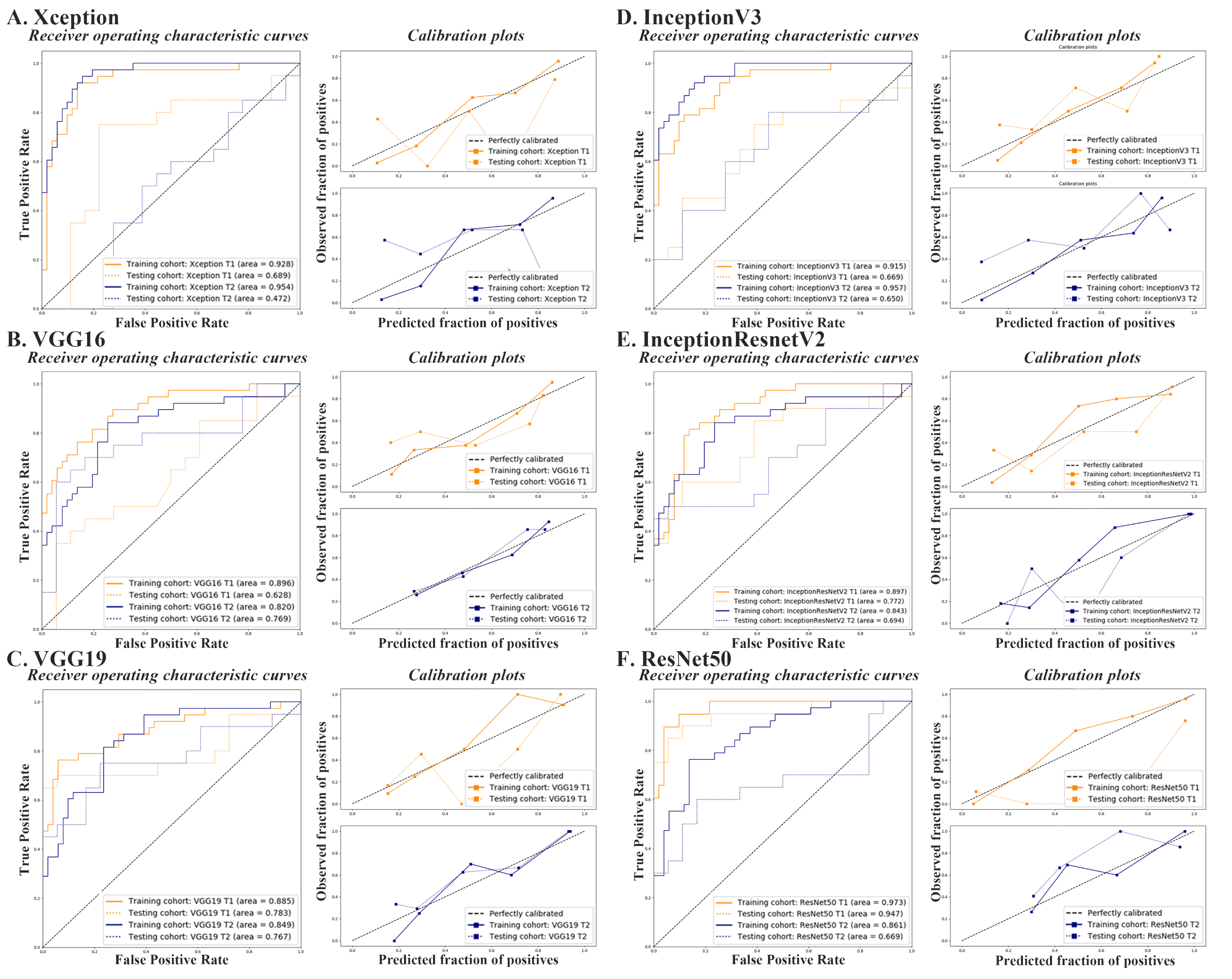


Evaluation of predictive performances for deep learning-based models using T1- or T2-derived MRI deep learning-associated features by ROC analysis and calibration analysis.

Abbreviations: DL, Deep learning; WDLPS, Well-differentiated liposarcoma; ROC, Receiver operating characteristic.

**Figure.S3. Evaluation of predictive performance of deep learning-based models on MRI- or CT-derived features in classification of WDLPS and lipoma on patients in the training and validation cohorts.**


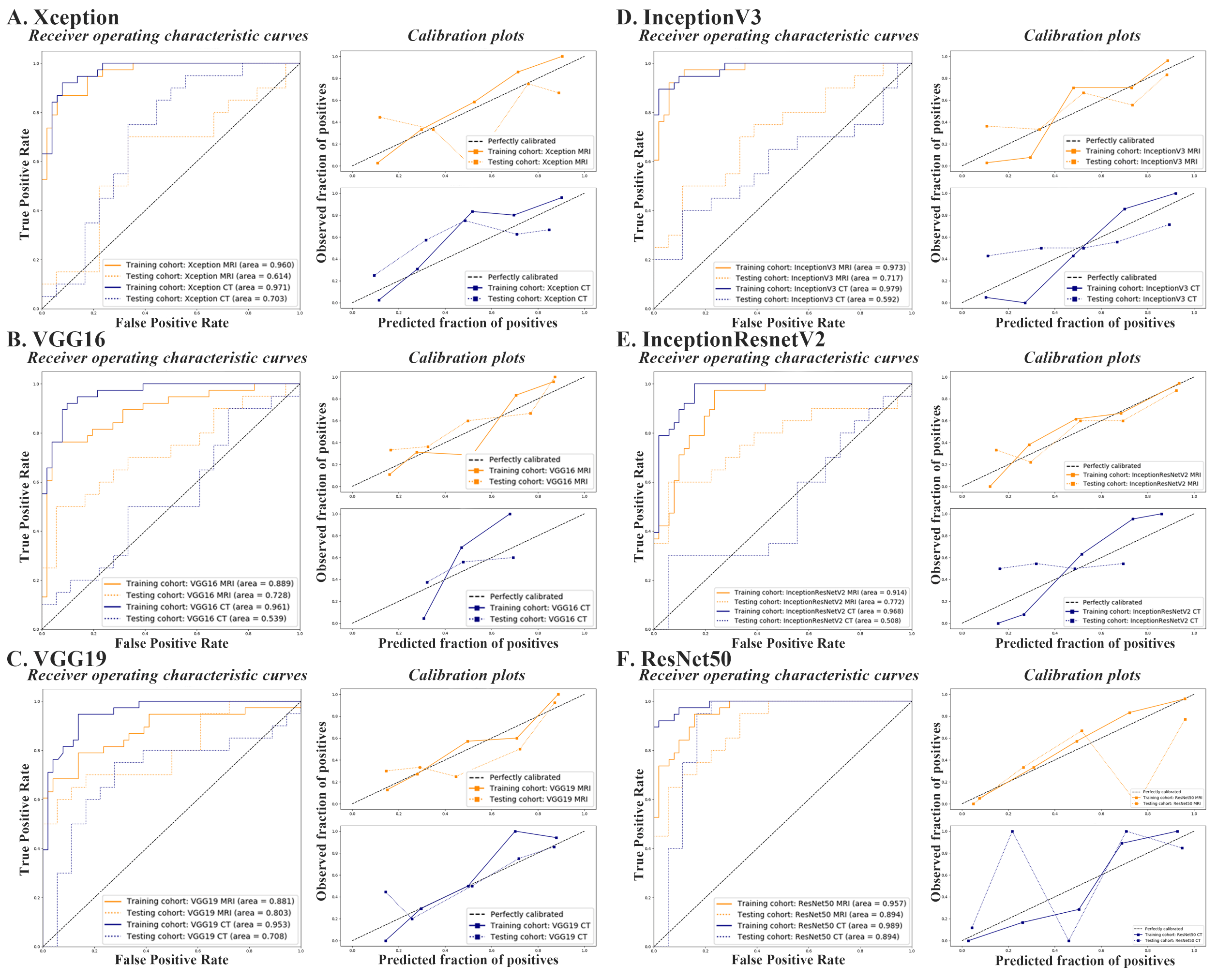


Evaluation of predictive performances for deep learning-based models using MRI- or CT-derived deep learning-associated features by ROC analysis and calibration analysis.

Abbreviations: DL, Deep learning; WDLPS, Well-differentiated liposarcoma; ROC, Receiver operating characteristic.

**Figure.S4. Evaluation of predictive performance of deep learning-based models on integrated multimodality MRI- and CT-derived features in classification of WDLPS and lipoma on patients in the training and validation cohorts.**


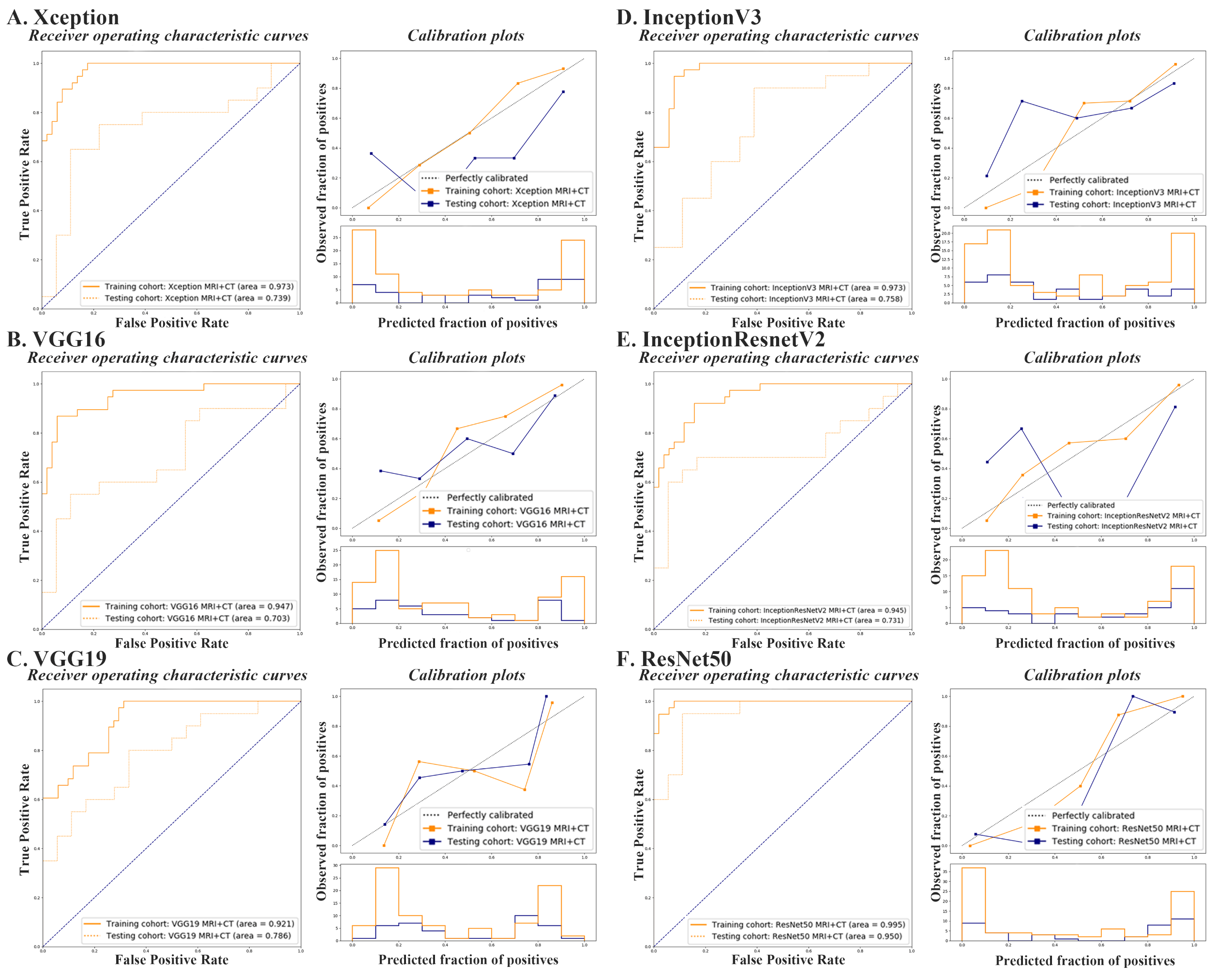


Evaluation of predictive performances for deep learning-based models integrating MRI- and CT-derived multimodality features by ROC analysis and calibration curves.

Abbreviations: DL, Deep learning; WDLPS, Well-differentiated liposarcoma; ROC, Receiver operating characteristic.

**Figure.S5. Evaluation of predictive performance of handcrafted radiomics models in classification of WDLPS and lipoma on patients in the training and validation cohorts.**


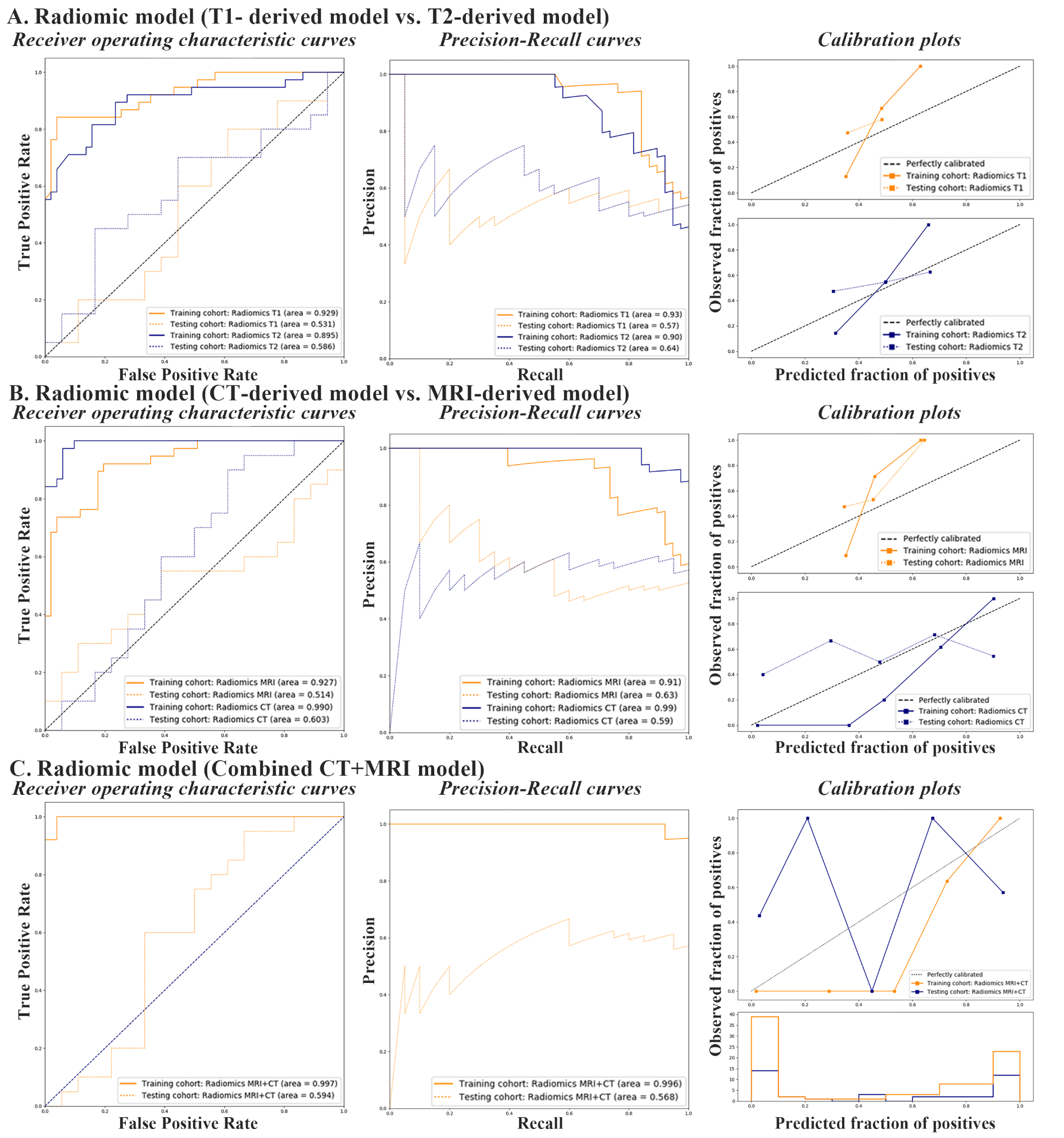


Evaluation of predictive performances for handcrafted radiomics models using CT- and MRI-derived handcrafted radiomic features by ROC analysis, precision-recall plots, and calibration analysis.

Abbreviations: WDLPS, Well-differentiated liposarcoma; ROC, Receiver operating characteristic.

**Reference**

1. Chollet F. Xception: Deep Learning with Depthwise Separable Convolutions. <https://arXivorg/abs/161002357>. 2017.

2. Karen Simonyan AZ. Very Deep Convolutional Networks for Large-Scale Image Recognition. <https://arXivorg/abs/14091556>. 2014.

3. He K, Zhang X, Ren S, et al., editors. Deep Residual Learning for Image Recognition. IEEE Conference on Computer Vision & Pattern Recognition; 2016.

4. Christian Szegedy VV, Sergey Ioffe, Jonathon Shlens, Zbigniew Wojna. Rethinking the Inception Architecture for Computer Vision. <https://arXivorg/abs/151200567>. 2015.

5. Christian Szegedy SI, Vincent Vanhoucke, Alex Alemi. Inception-v4, Inception-ResNet and the Impact of Residual Connections on Learning. <https://arxivorg/abs/160207261>. 2016.

6. Olga Russakovsky JD, Hao Su, Jonathan Krause, Sanjeev Satheesh, Sean Ma, Zhiheng Huang, Andrej Karpathy, Aditya Khosla, Michael Bernstein, Alexander C. Berg, Li Fei-Fei. ImageNet Large Scale Visual Recognition Challenge. <https://arxivorg/abs/14090575>. 2014.

7. van Griethuysen JJM, Fedorov A, Parmar C, et al. Computational Radiomics System to Decode the Radiographic Phenotype. Cancer research. 2017;77(21):e104-e7.

8. Zwanenburg A, Leger S, Vallières M, et al. Image biomarker standardisation initiative - feature definitions. 2016.

9. Peeken JC, Bernhofer M, Wiestler B, et al. Radiomics in radiooncology - Challenging the medical physicist. Physica medica : PM : an international journal devoted to the applications of physics to medicine and biology : official journal of the Italian Association of Biomedical Physics (AIFB). 2018;48:27-36.

10. Deist T, Dankers F, Valdes G, et al. Machine learning algorithms for outcome prediction in (chemo)radiotherapy: An empirical comparison of classifiers. Medical Physics. 2018;45.

11. Vickers AJ, Cronin AM, Elkin EB, et al. Extensions to decision curve analysis, a novel method for evaluating diagnostic tests, prediction models and molecular markers. BMC medical informatics and decision making. 2008;8:53.

12. Harrell F. [R-pkgs] New version of Hmisc package on CRAN.
